# Loss of SMURF2 expression enhances RACK1 stability and promotes ovarian cancer progression

**DOI:** 10.1038/s41418-023-01226-w

**Published:** 2023-10-12

**Authors:** Yanan Pi, Qiushi Feng, Fusheng Sun, Zhiqiang Wang, Yue Zhao, Dejia Chen, Yiming Liu, Ge Lou

**Affiliations:** 1 Department of Gynecology, Harbin Medical University Cancer Hospital, Harbin, 150086 P. R. China; 2 Heilongjiang Academy of Chinese Medical Sciences, Harbin, 150036 P. R. China

**Keywords:** Oncogenes, Genetics research

## Abstract

Receptor for activated C kinase 1 (RACK1) has been confirmed to take part in multiple biological events and the mechanism supporting abnormal RACK1 expression in ovarian cancer (OC) remains to be characterized. Here, we identified Smad ubiquitin regulatory factor 2 (SMURF2) as a bona fide E3 ligase of RACK1 in OC. SMURF2 effectively added the K6, K33 and K48 ubiquitin chains to the RACK1, resulting in polyubiquitination and instability of RACK1. PCAF promoted acetylation of RACK1 at K130, leading to SMURF2-mediated RACK1 ubiquitination inhibited and promote OC progression. The expression levels of SMURF2 and RACK1 were negatively correlated. SMURF2 was abnormal low expression in human ovarian cancer, resulting in decreased ubiquitination of RACK1 and increased stability, which promoted OC progression, and strongly associated with poor patients’ prognosis. In general, our results demonstrated that SMURF2 plays a pivotal role in stabilizing RACK1, which in turn facilitates tumorigenesis in OC, suggesting that SMURF2-RACK1 axis may prove to be potential targets for the treatment of OC.

## Introduction

Ovarian cancer (OC) is one of the most common cancers in the female genital tract, which is the third most common cancer behind cervical cancer and endometrial cancer, and the most deadly, being the 8th most common cause of cancer deaths among women [1]. The overall survival of OC patients has not improved significantly over the past decades due to the lack of typical and easily detectable early clinical signs, the lack of durable and effective treatment, and the elevated risk of chemoresistance and recurrence, with most cases being clinically advanced and with distant metastases at the time of diagnosis [2]. Therefore, in order to provide a theoretical basis for improving clinical outcomes in OC, there is an urgent need to better understand the potential pathogenesis of OC and to significantly improve our understanding of tumor progression and metastasis.

Post-translational modifications (PTMs) are a dynamic and reversible post-translational epigenetic modification of proteins with low metabolic cost and sensitive response [3]. It is an important mechanism for increasing the variety and functional diversity of proteins and plays a key role in regulating metabolism, signal transduction, reproductive development, tumor inflammation and other physiological and pathological conditions. Ubiquitination is an important type of PTMs, plays a crucial role in regulating the “quantity” and “quality” of substrate proteins, thus helping to ensure homeostasis of the intracellular environment and the smooth running of life processes. Malfunctions of the ubiquitin-proteasome system are responsible for more than 80% of proteins are degraded in cells and have been demonstrated to cause pathologies, particularly malignant tumors [4]. Ubiquitination is a cascading process in which ubiquitin, a universally expressed 76 amino acid protein, first binds to ubiquitin-activating enzymes (E1s) and activates ubiquitin, then transfers the activated ubiquitin molecule to ubiquitin-binding enzymes (E2s). Ubiquitin ligases (E3s) then transfer ubiquitin molecules from the E2s to the underlying protein, ultimately leading to its degradation by the proteasome. Due to their relative specificity in recognizing substrate proteins, E3 ligases play a key role in the overall ubiquitination process.

Smad ubiquitin regulatory factor 2 (SMURF2) is a HECT-type E3 ubiquitin (Ub) ligase that regulates many key functional proteins, including SATB1 [5], RNF20 [6], YY1 [7], and Smad2 [8], which are involved in oncogenic or tumor suppressor functions. Recent studies have shown that SMURF2 interacts with SIRT1 to mediate the degradation of SIRT1, while deletion of SMURF2 expression leads to upregulation of SIRT1, inducing tumor initiation and the invasive metastasis of colorectal cancer in vivo and in vitro [9]. In addition, SMURF2 induces enhanced tumor metastasis in nude mice models of breast cancer [10]. Nevertheless, the role of SMURF2 in the progression of OC has not yet been the subject of in-depth investigation.

The receptor for activated C-kinase 1 (RACK1) is involved in a wide range of cellular signaling pathways, as a member of the tryptophan-aspartate repeat (WD repeat) family of proteins. Identified as a classic scaffolding protein for a variety of kinases and receptors, RACK1 is involved in a number of intracellular signaling pathways and plays a key role in a variety of physiological processes including cell growth, migration and differentiation [11–15]. The function of RACK1 in tumor progression is tissue specific in different biological contexts. RACK1 is downregulated and has a suppressive role in gastric cancer [13]. In contrast, RACK1 is upregulated and promotes tumor progression in hepatocellular carcinoma [11, 12], breast cancer [16] and lung adenocarcinoma [17, 18]. The exact role of RACK1 in the progression and metastasis of OC is not yet known.

Both acetylation and ubiquitination occur on lysine residues in proteins and work together to control key cellular functions [19]. Acetylation controls a wide range of biological processes, including enzymatic activity, transcription and the subcellular localization of numerous proteins [20, 21]. Previous studies have shown that acetylation can also contribute to the stabilization or degradation of proteins through co-ordination with ubiquitination. For example, acetylation has been shown to compete fiercely with E3s/DUB for the same lysine residues, thereby co-regulating the stability of the target substrate protein [22]. Another potential mechanism by which acetylation-mediated protein degradation or stabilization is that acetylation can promote the formation or dissociation of E3s/DUBs-substrate complexes [23, 24]. We therefore speculate that acetylation may participate in regulating RACK1 ubiquitination and stability.

This study identified SMURF2 as a negative regulator of OC cell proliferation and invasion. Mechanistically, we showed that SMURF2 expression was downregulated in OC tissues, and SMURF2 could promote RACK1 ubiquitination and inhibite OC cell proliferation, migration and invasion. Furthermore, we found that RACK1 is acetylated by PCAF. The reduced interaction of SMURF2 with acetylated RACK1 may be a potential molecular mechanism for the acetylation-dependent stabilization of RACK1. In OC tissues, SMURF2 protein showed a strong negative correlation with RACK1 expression levels. Abnormally low expression of SMURF2 protein or high expression of RACK1 both predicted poor prognosis, suggesting that the SMURF2-RACK1 axis may prove to be an essential regulatory role in the pathogenesis and treatment of OC.

## Results

### SMURF2 interacts with RACK1

**Fig. 1:**
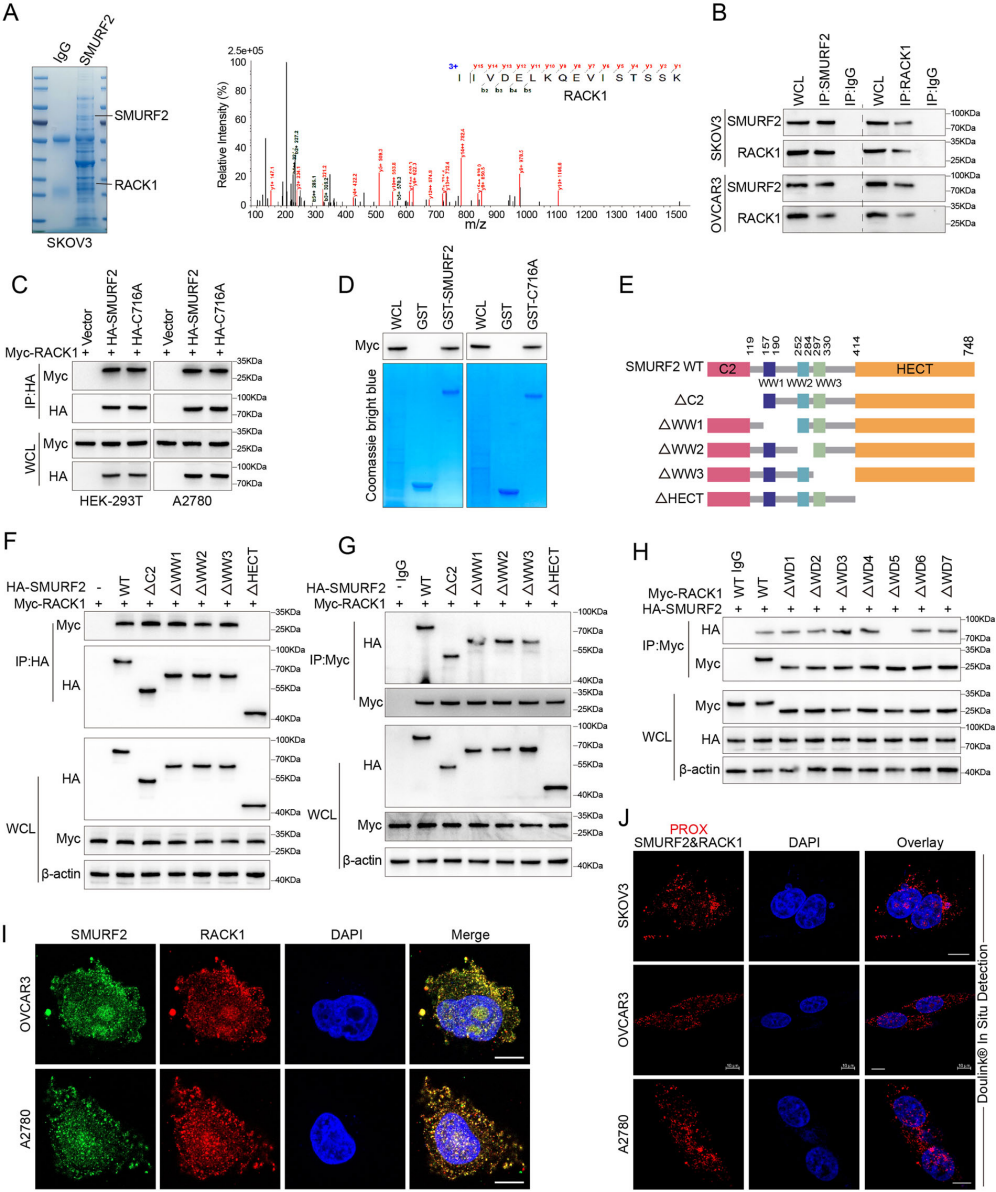
SMURF2 interacts with RACK1. **A** Mass-spectrometry analysis of an RACK1 peptide in SMURF2 precipitate. **B** Cell lysates from OVCAR3 and SKOV3 were analyzed by IP using antibodies against SMURF2 and RACK1, then subjected to IB analysis. IgG was used as the isotype control. **C** HEK293T and A2780 cells were transfected with Myc-RACK1 alone or in combination with HA-tagged SMURF2 WT or SMURF2 C716A, and cell lysates were analyzed by IP with HA beads followed by IB with antibodies against Myc and HA. **D** Purified Myc-tagged RACK1 was incubated with GST, GST-SMURF2 or GST-SMURF2 C716A coupled to glutathione-Sepharose beads. Proteins retained on Sepharose were then subjected to IB with indicated antibodies. Recombinant GST, GST-SMURF2 or GST-SMURF2 was purified from bacteria and analyzed by SDS-PAGE and Coomassie blue staining. **E** Schematic representation of HA-tagged full-length (FL) SMURF2 and its various deletion mutants. **F**, **G** HEK293T cells were cotransfected with Myc-RACK1 and HA-tagged FL SMURF2 or its deletion mutants, and cell lysates were analyzed by IP with HA(F) or Myc(G) beads followed by IB with antibodies against Myc and HA. **H** HEK293T cells were cotransfected with HA-SMURF2 and Myc-tagged FL RACK1 or its deletion mutants, and cell lysates were analyzed by IP with Myc beads followed by IB with antibodies against HA and Myc. **I** Confocal images showing colocalization of SMURF2 (green) and RACK1 (red) in OVCAR3 and A2780. Nuclei were counterstained with DAPI (blue). **J** In situ PLA between endogenous SMURF2 and RACK1 in OVCAR3, SKOV3 and A2780 cells. Representative images are shown with merged PLA and nuclei (DAPI) channels from PLA experiments. Scale bars: 10 μm (**I** and **J**).

In order to investigate the potential role of SMURF2 in OC, we analysed the results of RNA sequencing from The Cancer Genome Atlas (TCGA) and the GTEx database. We found that abnormally low expression of the SMURF2 transcript in OC tissues was associated with poorer survival in patients (Supplementary Fig. 1A). These findings suggest SMURF2 may play a key role in OC progression. To identify potential downstream targets of SMURF2, we performed mass spectrometry analysis of proteins interacting with SMURF2 in OC cells (Supplementary Table 1). Combined with the biological functions of the proteins, we first identified the potential downstream proteins of SMURF2 such as RACK1, EIF3c and eIF3a (Fig. 1A). To further identify the SMURF2 target protein, we knocked down SMURF2 in OVCAR3 cells. SMURF2 knockdown increased the protein levels of RACK1, but had a limited effect on the protein levels of EIF3c and eIF3a, suggesting that RACK1 is a promising candidate target protein (Supplementary Fig. 1B). We confirmed that endogenous SMURF2 and RACK1 could co-precipitate in A2780, OVCAR3 and SKOV3 cells (Fig. 1B, Supplementary Fig. 1C). Consistent with the results observed for endogenous immunoprecipitation (IP), ectopically expressed Myc-tagged RACK1 could be detected in HA-tagged SMURF2 and vice versa (Supplementary Fig. 1D, E). Co-IP assay showed that Myc tagged RACK1 was readily detected in HA-SMURF2 WT (wild-type) or HA-SMURF2 C716A IP from HEK293T cells and A2780 (Fig. 1C), suggesting that SMURF2 enzyme activity was not required for SMURF2 to bind to RACK1. We also performed in vitro GST pull-down assays by mixing purified Myc-RACK1 with purified recombinant GST-SMURF2 or GST-SMURF2 C716A proteins. As shown in Fig. 1D, either SMURF2 or its SMURF2 C716A mutant binds to Myc-RACK1, but GST did not bind to Myc-RACK1 alone, confirming that the interaction of SMURF2 with RACK1 was straightforward. To map the minimum necessary region for their interaction, we constructed a series of shortened mutants of HA-SMURF2 and Myc-RACK1 (Fig. 1E, Supplementary Fig. 1F). This allowed us to narrow down the range of binding sites. Truncated mutation analysis revealed that the HECT domain of SMURF2 and the WD5 domain of RACK1 are both necessary and sufficient for direct interactions (Fig. 1F–H, Supplementary Fig. 1G). Immunofluorescence (IF) staining revealed that in OC cells, endogenous SMURF2 and RACK1 predominantly co-localize in the nucleus and cytoplasm, and in HEK-293T cells, exogenous SMURF2 and RACK1 also predominantly co-localize in the nucleus and cytoplasm (Fig. 1I, Supplementary Fig. 1H). In addition, using in situ proximity ligation assays (PLA), we detected interactions between endogenous SMURF2 and RACK1 in OVCAR3, A2780 and SKOV3 cells (Fig. 1J). Collectively, these findings suggest that RACK1 is a bona fide SMURF2-interacting protein.

### SMURF2 negatively regulates RACK1 stability

**Fig. 2:**
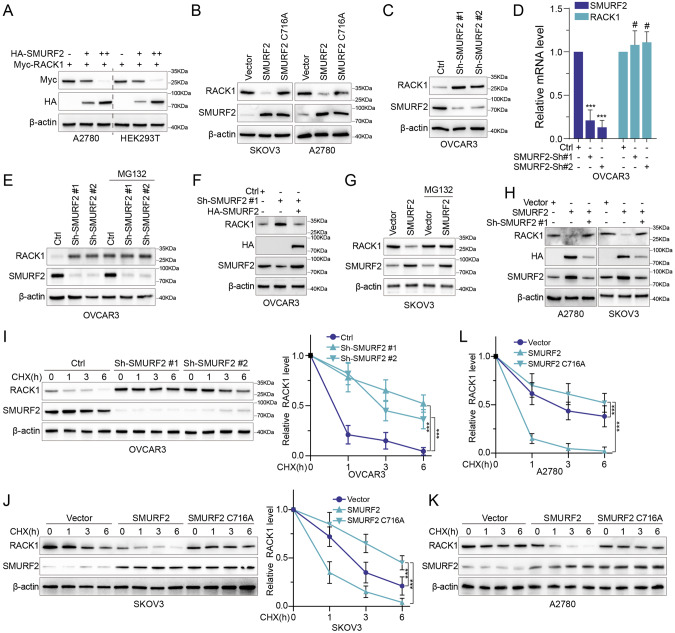
SMURF2 negatively regulates RACK1 stability. **A** HEK293T and A2780 cells were co-transfected with an increasing amount of HA-tagged SMURF2 and a constant amount of Myc-RACK1. The cell lysates were analyzed by IB with anti-Myc antibody. **B** SMURF2 WT or C716A mutant was overexpressed in SKOV3 and A2780 cells. The protein levels of RACK1 were analyzed. **C** SMURF2 was knocked down in OVCAR3 cells using two independent shRNAs. The protein levels of RACK1 were analyzed by IB. **D** In OVCAR3 cells, mRNA levels of RACK1 were analyzed by qRT-PCR after SMURF2 was knocked down using two independent shRNAs. **E** OVCAR3 cells transfected with 2 independent SMURF2 shRNA were treated with or without the proteasome inhibitor MG132 (20 μM, 8 h), and then SMURF2 and RACK1 were analyzed. **F** IB analysis of RACK1 levels in OVCAR3 cells transduced with SMURF2 shRNA, together with either HA-vector or HA-SMURF2. **G** SMURF2 was ectopically expressed in SKOV3 cells, and SKOV3 cells were treated with or without the proteasome inhibitor MG132 (20 μM, 8 h), and then analyzed for SMURF2 and RACK1. **H** IB analysis of RACK1 levels in SKOV3 and A2780 cells transduced with SMURF2 ectopically, together with either sh-Control or sh-SMURF2 #1. **I** OVCAR3 cells stably expressing control shRNA or SMURF2 shRNA were treated with 100 μg/ml CHX, harvested at the indicated times, and then subjected to IB with antibodies against RACK1 and SMURF2. Quantification of RACK1 levels relative to β-actin is shown. **J** SKOV3 cells were transfected with SMURF2 or SMURF2 C716A, treated with 100 μg/ml CHX, collected at the indicated times, and then subjected to IB with antibodies against SMURF2 and RACK1. Quantification of RACK1 levels relative to β-actin is shown. **K**, **L** A2780 cells were transfected with SMURF2 or SMURF2 C716A, treated with 100 μg/ml CHX, collected at the indicated times, and then subjected to IB with antibodies against SMURF2 and RACK1. Quantification of RACK1 levels relative to β-actin is shown. Data are represented as mean ± SD of 3 independent experiments. ***P < 0.001, # No statistically significance, 1-way ANOVA with Dunnett’s post test (**I**, **J** and **L**).

The interaction of SMURF2 with RACK1 suggests that SMURF2 is the E3 ligase that targets RACK1 for degradation. To further confirm this, we overexpressed SMURF2 in OC cells and HEK293T, found that ectopic increased expression of SMURF2 resulted in a dose-dependent decrease in RACK1 (Fig. 2A). Next, we investigated whether SMURF2 enzymatic activity was involved in the reduction of RACK1 levels. We overexpressed SMURF2 WT or the catalytically inactivated mutant SMURF2 C716A in SKOV3 and A2780 cells (Supplementary Fig. 2A). As shown in Fig. 2B, SMURF2 WT, but not the C716A mutant, reduced RACK1 levels. In contrast, RACK1 accumulated in OVCAR3 cells when SMURF2 expression was knocked down with two different shRNAs (Fig. 2C). However, deletion or overexpression of SMURF2 had no significant effect on RACK1 mRNA levels (Fig. 2D, Supplementary Fig. 2B). We found that the absence of SMURF2 significantly increased the expression of RACK1, while the addition of the proteasome inhibitor MG132 or overexpression of HA-SMURF2 could almost completely reverse the effect (Fig. 2E–G). The autophagy inhibitor CQ had no such effect (Supplementary Fig. 2C). In addition, the reduction in RACK1 levels caused by SMURF2 overexpression was almost completely reversed by SMURF2 shRNA#1 (Fig. 2H). We used cyclohexanone (CHX) to block protein synthesis and detected RACK1 levels after interfering with SMURF2 expression to demonstrate that SMURF2 itself affects RACK1 stability. Forced expression of SMURF2 in A2780 and SKOV3 cells, but not the C716A mutant, resulted in a significant decrease in RACK1 stability (Fig. 2I, J), whereas SMURF2 knockdown in OVCAR3 resulted in a significant increase in RACK1 stability (Fig. 2K, L). Taken together, these results suggest that SMURF2 is a specific regulator of RACK1 stability.

### SMURF2 is an E3 ubiquitin ligase for RACK1

**Fig. 3:**
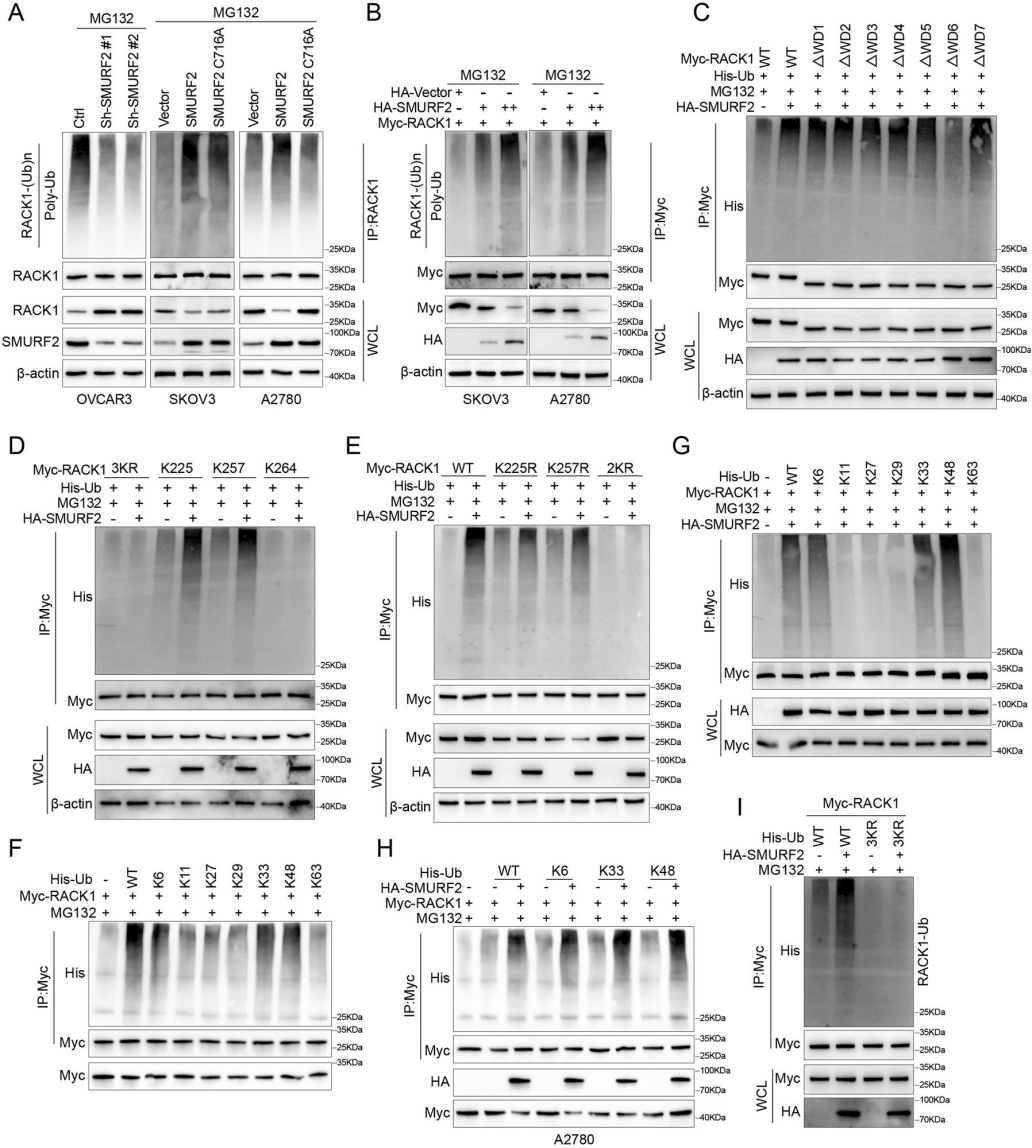
SMURF2 is an E3 ubiquitin ligase for RACK1. **A** OVCAR3 cells were transfected with the indicated ShRNAs (left panel), SKOV3 and A2780 cells were transfected with SMURF2 WT or SMURF2 C716A plasmids (middle and right panels). Cell lysates were subjected to IP with RACK1 antibody, followed by IB with antibodies against Ub and RACK1. Cells were treated with 20 μM MG132 for 8 h before harvesting. **B** SKOV3 or A2780 cells were cotransfected with an increasing amount of HA-tagged SMURF2 and a constant amount of Myc-RACK1, and cell lysates were subjected to IP with Myc beads followed by IB with antibodies against Myc and Ub. Cells were treated with 20 μM MG132 for 8 h before harvesting. **C** Ubiquitination assay of Myc-tagged FL RACK1 and its various deletion mutants in HEK293T cells cotransfected with His-Ub and HA-SMURF2, and treated with 20 μM MG132 for 8 h. **D** Ubiquitination assay of RACK1 in HEK293T cells cotransfected with His-Ub, HA-SMURF2, Myc-RACK1 3KR, Myc-RACK1-225K, Myc-RACK1-257K and Myc-RACK1-264K and treated with 20 μM MG132 for 8 h. **E** Ubiquitination assay of RACK1 in HEK293T cells cotransfected with His-Ub, HA-SMURF2, Myc-RACK1 WT, Myc-RACK1- K 225 R, Myc-RACK1- K 257 R and Myc-RACK1-2KR and treated with 20 μM MG132 for 8 h. **F** RACK1 poly-ubiquitination linkage was examined by transfecting His-tagged WT or indicated ubiquitin mutants containing Lys 6/11/27/29/33/48/63-only mutations (the other six of seven lysine residues were mutated to arginine) together with Myc-RACK1 into HEK293T cells, followed by IB analysis of His-Ub in anti-Myc IP products. Cells were treated with 20 μM MG132 for 8 h. **G** Effects of SMURF2 overexpression on RACK1 poly-ubiquitination in HEK293T cells transfected with the indicated ubiquitin Lys 6/11/27/29/33/48/63-only mutant plasmids. A total of 36 h after transfection, cells were treated with 20 μM MG132 for 8 h before harvesting for Myc-tag IP and His-Ub IB analyses. **H** Effects of SMURF2 overexpression on RACK1 poly-ubiquitination in A2780 cells transfected with the indicated ubiquitin Lys 6/33/48-only mutant plasmids. A total of 36 h after transfection, cells were treated with 20 μM MG132 for 8 h before harvesting for Myc-tag IP and His-Ub IB analyses. **I** RACK1 poly-ubiquitination linkage was examined by transfecting His-tagged WT ubiquitin or His-tagged WT 3KR (Lys 6/33/48 were all mutated to arginine) ubiquitin together with Myc-RACK1 into HEK293T cells, followed by IB analysis of His-Ub in anti-Myc IP products. Cells were treated with 20 μM MG132 for 8 h.

We ectopically expressed SMURF2 or C716A mutants in SKOV3 and A2780 cells to investigate whether SMURF2 actually catalyzes the ubiquitination of RACK1. After RACK1 IP in cells treated with MG132, we observed that RACK1 was highly ubiquitinated. In SKOV3 and A2780 cells, ectopic expression of SMURF2, but not the C716 mutant, significantly increased polyubiquitination of RACK1. In contrast, RACK1 ubiquitination was significantly reduced by downregulating SMURF2 expression with two independent shRNAs (Fig. 3A). Meanwhile, SMURF2 could induce the RACK1 protein to exhibit a pronounced, gradually increasing level of ubiquitination with increasing SMURF2 concentration (Fig. 3B). Next, to determine which lysine sites on RACK1 are involved in SMURF2-mediated ubiquitination, we generated different RACK1 fragments according to the protein domain and found that RACK1 fragments containing the WD6 domain can be modified by SMURF2-mediated ubiquitination (Fig. 3C). Furthermore, we mutated all other lysines in the WD6 domain to arginine while retaining only one lysine. It was found that Lys-225 and Lys-257 may be the key sites for SMURF2-mediated ubiquitination of RACK1 (Fig. 3D, E).

Ubiquitination controls protein stability, transport and enzyme activity through seven types of linkages between ubiquitin molecules. Therefore, we sought to determine the type of ubiquitin chain that SMURF2 adds to RACK1. To determine the ubiquitin chain type of RACK1 in cells ectopically expressing SMURF2, we mutated each lysine in ubiquitin (e.g., K48R) or six of the seven lysines to arginine (e.g., K48). The results showed that SMURF2 can effectively add K6, K33, and K48 ubiquitin chains to RACK1 (Fig. 3F, Supplementary Fig. 3B). To extend our results, we performed a comprehensive in vitro ubiquitination assay on RACK1 with SMURF2 based on a series of ubiquitin mutants, and the results showed that SMURF2 effectively increased K6, K33, and K48 ubiquitin chains on RACK1 in vitro (Fig. 3G, H, Supplementary Fig. 3A).When we simultaneously mutated K6, K33, and K48 in the ubiquitin molecule to arginine (His-Ub 3KR), we found no significant increase in RACK1 ubiquitination in cells with high ectopic expression of SMURF2 (Fig. 3I). In conclusion, our study proposes that SMURF2 is a specific E3 ligase that perturbs the stability of RACK1.

### RACK1 acetylation inhibits interaction with SMURF2, resulting in RACK1 stabilization

**Fig. 4:**
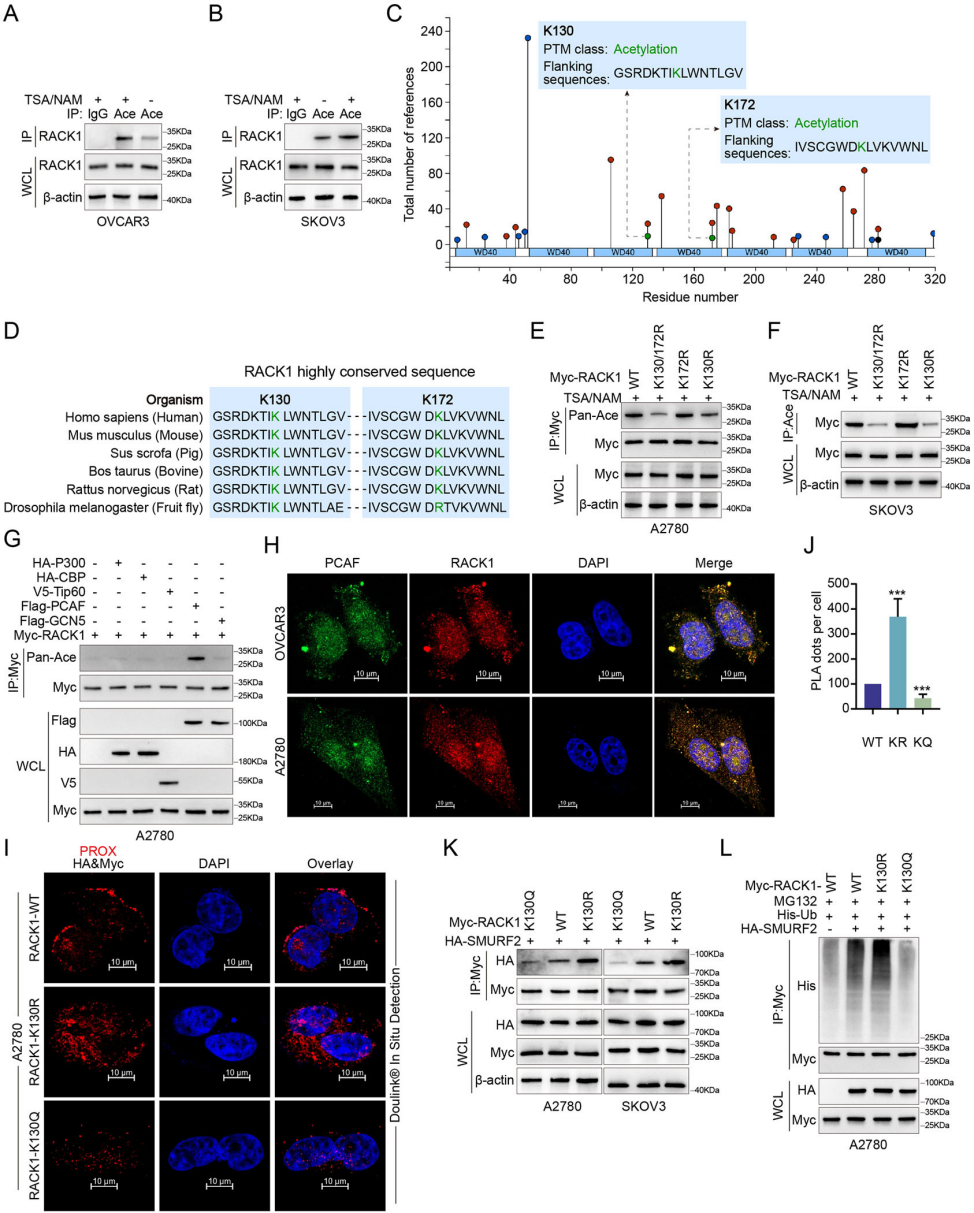
RACK1 acetylation inhibits interaction with SMURF2, resulting in RACK1 stabilization. **A**, **B** NAM (10 mM, 4 h) and TSA (0.2 μM, 4 h) were used to improve protein acetylation levels, and acetylated RACK1 in OVCAR3 and SKOV3 cells was immunoprecipitated with anti-Pan Ace antibodies. **C** Analysis of proteomic databases indicates that 2 lysine residues of RACK1 are potentially acetylated. **D** Sequence alignment of the conserved K130 and K172 containing region in RACK1 orthologs of different species. **E** A2780 cells were transfected with Myc-RACK1 WT, Myc-RACK1 K130/172 R, Myc-RACK1 K172R and Myc-RACK1 K130R. NAM (10 mM, 4 h) and TSA (0.2 μM, 4 h) were used to improve protein acetylation levels, and acetylated RACK1 in A2780 cells was IP with anti-Myc antibodies and IB with anti-Pan Ace antibodies. **F** SKOV3 cells were transfected with Myc-RACK1 WT, Myc-RACK1 K130/172 R, Myc-RACK1 K172R and Myc-RACK1 K130R. NAM (10 mM, 4 h) and TSA (0.2 μM, 4 h) were used to improve protein acetylation levels, and acetylated RACK1 in SKOV3 cells was IP with anti-Pan Ace antibodies and IB with anti-Myc antibodies. **G** A2780 were co-transfected with HA-P300, Flag-GCN5, V5-Tip60, HA-CBP, or Flag-PCAF and Myc-RACK1, and RACK1 acetylation levels were determined by IP with anti-Pan Ace antibodies. **H** The confocal image above shows PCAF (green) and RACK1 (red) co-located in OVCAR3 and A2780 cells. **I**, **J** In situ PLA between exogenous HA-SMURF2 and Myc-RACK1 WT, Myc-RACK1 K130R or Myc-RACK1 K130Q in A2780 cells. Representative images are shown with merged PLA and nuclei (DAPI) channels from PLA experiments. Scale bars: 10 μm (**I**). **K** A2780 and SKOV3 cells were co-transfected with Myc-RACK1 WT, Myc-RACK1 K130R or Myc-RACK1 K130Q and HA-SMURF2, and cell lysates were analyzed by IP with Myc beads followed by IB with antibodies against Myc and HA. **L** Ubiquitination assay of RACK1 in A2780 cells cotransfected with His-Ub, HA-SMURF2, Myc-RACK1 WT, Myc-RACK1-K130R and Myc-RACK1- K130Q and treated with 20 μM MG132 for 8 h. **A**, **B**, **E**–**I**, **K**, **L** Results are representative of three independent experiments, J The data are shown as mean ± s.d. One-way ANOVA test, ***p < 0.001.

Several studies have shown that acetylation of oncogenic proteins generally increases their protein stability, resulting in increased oncogenic activity. We found that the acetylation level of RACK1 was increased by the combined application of trichostatin A (TSA) and nicotinamide (NAM) (Fig. 4A, B). Analysis of the proteomic database revealed that two potential acetylation sites, Lys130 and Lys172 (https://www.phosphosite.org/), were present on RACK1 and highly conserved between species (Fig. 4C, D). To further clarify the lysine sites on RACK1 where acetylation modifications occur, we constructed a RACK1 single site mutant plasmid (Myc-RACK1 K130R and K172R) and a double site mutant plasmid (Myc-RACK1 K130/172 R). It was shown that the acetylation of RACK1 occurs mainly at the Lys130 site (Fig. 4E, F). P300 (E1A binding protein, 300 kDa), CREB binding protein (CBP), Tip60, P300/CBP-associated factor (PCAF) and GCN5 (KAT2A) had been identified as the major acetyltransferases in mammalian cells. The five acetyltransferases were cotransfected with RACK1 to determine the acetyltransferase of RACK1. The acetylation level of RACK1 could only be significantly increased by overexpression of PCAF. (Fig. 4G). IF staining also confirmed the co-localization of PCAF and RACK1 mainly in the nucleus and cytoplasm (Fig. 4H). Next, we ectopically expressed RACK1 WT, a simulated acetylated K130Q mutant or an acetylation-deficient K130R mutant in SKOV3 cells. In situ proximity ligation (PLA) and IP experiments showed that the interaction between RACK1 K130Q and SMURF2 was significantly reduced compared to RACK1 WT (Fig. 4I, J). In contrast to RACK1 WT, ectopic expression of RACK1 130Q effectively reversed the destabilizing effects mediated by SMURF2 (Fig. 4K). To determine the critical role of RACK1 acetylation at K130 in RACK1 renewal, cellular ubiquitination analysis and CHX-chase assays were performed. The results showed increased stability of the acetylated mimetic K130Q mutant protein (Fig. 4L, Supplementary Fig. 4A, B). Ectopic expression of Flag-PCAF resulted in increased stability of endogenous RACK1 in SKOV3 cells (Supplementary Fig. 4C). These results indicate that PCAF-mediated acetylation of RACK1 at K130 may lead to a decrease in SMURF2-mediated ubiquitination and an increase in RACK1 stability.

### Loss of SMURF2 promotes ovarian tumorigenesis via the upregulation of RACK1

**Fig. 5:**
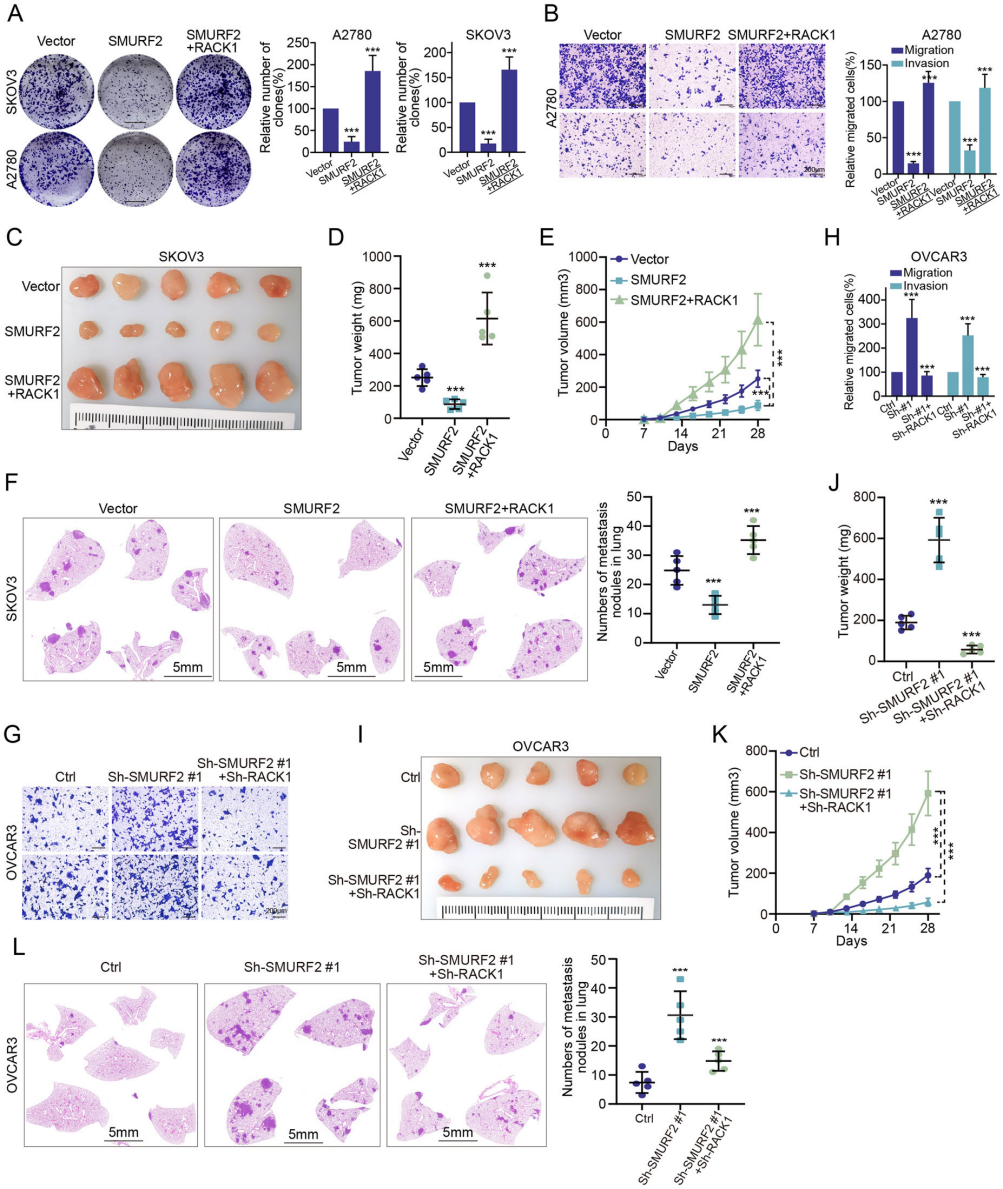
SMURF2 depletion promotes ovarian tumorigenesis via the upregulation of RACK1. **A**, **B** RACK1 was overexpressed ectopic in SKOV3 or A2780 cells with or without high SMURF2 expression. Cell growth and migration were examined by colony formation (**A**) and transwell assay (**B**). Scale: 1 cm (**A**); 200 μm (**B**). **C**–**E** The cotransfected SKOV3 cells were inoculated into the groin area of nude mice. Representative images of xenograft tumors are shown in **C**, and the xenograft weight (**D**) and growth curves for each group are calculated (**E**). Scale bars, 1 cm (**C**). **F** The co-transfected SKOV3 cells were injected intravenously into nude mice (*n* = 5 for each group). The representative HE staining of the lungs was shown in **F**, and the metastasis nodes of each group were calculated. Scale: 5 mm (**F**). **G**, **H** RACK1 was knocked down in OVCAR3 cells with low SMURF2 expression. Cell migration was examined by transwell assay (**G**) and the number of migrated cells in each group was counted (**H**). Scale: 200 μm (**G**). **I**–**K** The cotransfected OVCAR3 cells were inoculated into the groin area of nude mice. Representative images of xenograft tumors are shown in **I**, and the xenograft weight (**J**) and growth curves for each group are calculated (**K**). Scale bars, 1 cm (**I**). **L** The co-transfected OVCAR3 cells were injected intravenously into nude mice (*n* = 5 for each group). The representative HE staining of the lungs was shown in **L**, and the metastasis nodes of each group were calculated. Scale: 5 mm (**L**). **A**, **B**, **H** Results are representative of three independent experiments. **D**–**F**, **J**–**L** Tumor weight, tumor burden and metastasis nodule were analyzed. **A**, **B**, **D**–**F**, **H**, **J**–**L** Results are representative of three independent experiments, the data are shown as mean ± s.d. One-way ANOVA test, ***p < 0.001.

Previous studies have shown that RACK1 is a powerful tumor-promoting factor in OC [25], so we investigated whether SMURF2 inhibited the occurrence of ovarian tumors by regulating RACK1. To this end, we ectopically overexpressed RACK1 in SKOV3 and A2780 cells with and without SMURF2 overexpression. Ectopic SMURF2 expression significantly inhibited OC cell proliferation and migration, and restoration of RACK1 expression completely reversed the phenotype induced by ectopic SMURF2 expression (Fig. 5A, B, Supplementary Fig. 5A, B). In xenograft experiments, it was consistently demonstrated that RACK1 overexpression reversed the effects of ectopic SMURF2 expression and promoted tumor development and metastasis (Fig. 5C–F). In contrast, SMURF2 downregulation significantly promoted OVCAR3 cell proliferation and migration in vitro and in vivo, an effect that could be reversed by RACK1 knockdown (Fig. 5G–L, Supplementary Fig. 5C). Collectively, these results are consistent with the notion that RACK1 is a functional effector of SMURF2 in cancer.

### Reduced SMURF2 positively correlates with the upregulation of RACK1 and predicts a poor prognosis of OC patients

**Fig. 6:**
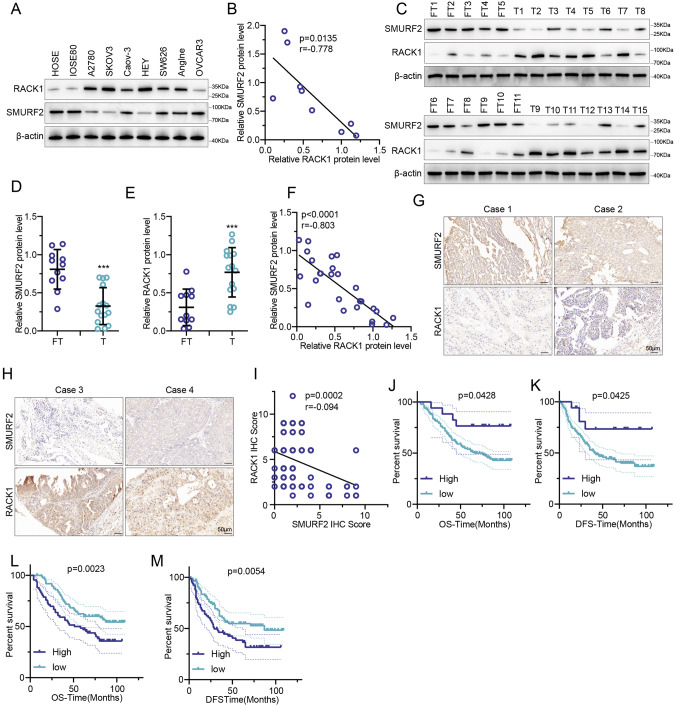
Reduced SMURF2 positively correlates with the upregulation of RACK1 and predicts a poor prognosis of OC patients. **A** Cell lysates from several OC cell lines were blotted with SMURF2 and RACK1 antibodies. **B** Correlation analysis of SMURF2 and RACK1 in OC cells. The Pearson *r* indicates correlation coefficient. **C**–**E** Protein expression levels of SMURF2 and RACK1 were compared in 11 FT tissues and 15 OC tissues, respectively. Cell lysates from 11 FT tissues and 15 OC tissues were blotted with SMURF2 (**D**) and RACK1 (**E**) antibodies. **F** Correlation analysis of SMURF2 and RACK1 in 11 FT tissues and 15 OC tissues. **G**, **H** Representative images of immunohistochemical staining of SMURF2 and RACK1 on tissue microarray of ovarian cancer specimens (*n* = 135). Scale bars are indicated. **I** SMURF2 expression correlates with RACK1 levels in tissue microarray of ovarian cancer samples. Protein levels of SMURF2 and RACK1 were quantified in ovarian cancer specimens. **J**, **K** Kaplan–Meier curves showing overall survival (OS) (**J**) and the disease-free survival (DFS) (**K**) of OC patients divided based on SMURF2 expression (*P* values by log-rank test). **L**, **M** Kaplan–Meier curves showing OS (**L**) and DFS (**M**) of OC patients divided based on RACK1 expression (*P* values by log-rank test). Data are represented as mean ± s.d of 3 independent experiments. ***P < 0.001, 2-tailed Student’s *t* test (**D**, **E**).

We hypothesized that downregulation of SMURF2 would lead to decreased ubiquitination and increased stability of RACK1, which may promote OC cell proliferation and migration. To test our hypothesis, we examined the correlation between the expression levels of SMURF2 and RACK1 in human OC cells and specimens. As shown in Fig. 6A, B, SMURF2 showed a negative correlation with RACK1 levels in OC cell lines (*P* = *, Pearson *r* = **). Similarly, we observed a negative correlation (*P* = **, Pearson *r* = **) between SMURF2 and RACK1 levels in 11 normal fallopian tube tissues and 15 OC tissues (Fig. 6C–F). We then performed immunohistochemical (IHC) staining for SMURF2 and RACK1 in tissue microarrays containing a cohort of OC samples (*n* = 138). Representative images of SMURF2 and RACK1 staining were shown in Fig. 6G, H. Low expression of SMURF2 and overexpression of RACK1 were observed in the majority of OC samples, with a significant negative correlation between SMURF2 and RACK1 (*P* < **, Pearson *r* = **) (Fig. 6I). SMURF2 downregulation was associated with RACK1 upregulation in OC tissues. Combined with information on the corresponding clinical survival prognosis of OC samples, we found that either low SMURF2 expression or elevated RACK1 expression was strongly associated with poor prognosis (overall survival or disease-free survival) of OC patients (Fig. 6J–M).

## Discussion

RACK1 is unstable, and there is evidence that post-translational modification is one of the major regulatory mechanisms governing the biological function of RACK1. It was previously reported that O-GlcNA acylation of RACK1 at serine 122 enhances its stability, ribosome localization and interaction with the protein kinase PKCβII, thereby promoting oncogenic translation and progression of hepatocellular carcinoma [12]. In this study, we provided experimental evidence that the E3 ligase SMURF2 played a key role in the ubiquitination and subsequent degradation of the oncogenic protein RACK1. Although the interaction between SMURF2 and RACK1 has not been previously reported, we clearly showed in the manuscript that SMURF2 directly bound to RACK1 through its HECT domain. In addition, we found that the acetyltransferase PCAF promoted acetylation of RACK1 at K130, leading to reduced ubiquitination and increased stability of RACK1. Mechanistically, RACK1 acetylation leads to reduced interaction with SMURF2, thereby inhibiting SMURF2-mediated RACK1 ubiquitination and promoting OC progression.

RACK1 has emerged as a potential prognostic marker and drug target in numerous cancers [11, 14, 26–28]. We and others had identified RACK1 as a key driver of the malignant biology of multiple tumors, including breast cancer [27], non-small cell lung cancer[14, 17] and OC [25]. RACK1 promotes cervical cancer invasion and lymph node metastasis through galectin-1 [29]. Our work provided sufficient experimental evidence that SMURF2 could also directly control the stability of RACK1 through post-translational modification. Specifically, abnormally low expression of the E3 ligase SMURF2 led to a decrease in the SMURF2-RACK1 complex, resulting in decreased ubiquitination of RACK1 and increased stability, which promoted tumor progression. By evaluating clinicopathological tissue microarrays, we demonstrated a significant negative correlation between SMURF2 and RACK1 expression levels. Therefore, our present study provides a potential molecular mechanism to explain the pathological elevation of RACK1.

Protein dynamics and highly conserved ubiquitination is a tightly controlled biological process, has become a key mechanism in many pathological processes. All seven lysine residues on ubiquitin can be modified to link multiple ubiquitin chains to protein substrates to maintain protein homeostasis [30]. In particular, all non-K63 ubiquitin chains can be targeted to promote protein degradation, supporting the idea that non-K63 ubiquitin chains are critical for the function of the ubiquitin-proteasome system [31]. Fbxo45 activates ERK activity in lung cancer through NP-STEP46 degradation mediated by K6 linkage to ubiquitin chains [32]. SMURF2 acted as a specific E3 ligase that effectively adds the K6, K33 and K48 ubiquitin chains to the RACK1, resulting in polyubiquitination and instability of RACK1. Conserved K48 ubiquitin chains tend to degrade target proteins. Based on the results of this study, we propose that SMURF2, an E3 ligase that is aberrantly expressed in OC tissues, leads to a reduction in its mediated ubiquitination of K6, K33 and K48 on the RACK1, which in turn increases the stability of RACK1 and thus promotes OC progression.

Crosstalk between the two lysine modifications (acetylation and ubiquitination) affects the stability and activity of the protein [19, 20]. We first demonstrated that SMURF2-mediated polyubiquitination of the RACK1 occurred at Lys225 and Lys257, whereas PCAF-mediated acetylation of RACK1 occurred at Lys130. Ubiquitination and acetylation of RACK1 do not compete for the same lysine site, so competition for the same lysine site does not yet explain the potential mechanism for acetylation-dependent stabilization of RACK1. On the other hand, we observed that SMURF2 preferentially bound to RACK1 K130R compared to RACK1 WT or K130Q, suggesting that acetylation may be a signaling switch that regulates RACK1 function by modulating the affinity of RACK1 for its interacting proteins, including SMURF2. Although the detailed molecular mechanism requires further analysis, our findings provide a mechanistic explanation for the signaling dependence of the RACK1-SMURF2 interaction.

In conclusion, we have identified SMURF2 as the E3 ligase of RACK1. In addition, our results suggest that the abnormally low expression of SMURF2 in OC tissues and the PCAF-mediated acetylation of RACK1 undoubtedly further impairs the ubiquitin degradation of RACK1 by SMURF2. While it is clear that RACK1 expression can be regulated at the transcriptional level, our data suggest that decreased SMURF2-mediated degradation of RACK1 through the ubiquitin-proteasome pathway is another important mechanism for abnormally high RACK1 expression in human OC. Given that abnormally low SMURF2 expression is responsible for elevated RACK1 levels and ovarian tumor progression, our results suggest that targeting RACK1 in patients with abnormally low SMURF2 expression may be a viable stratified treatment approach. In future work, it will be crucial to screen and identify SMURF2-specific agonists that activate SMURF2 activity in cancer cells to inhibit RACK1-dependent OC progression.

## Materials and methods

### Antibodies and reagents

Antibodies were purchased from Cell Signaling Technology against the following proteins. HA (1:2000, 3724), Myc (1:2000, 2276), His (1:2000, 12698), ubiquitin (1:1000, 20326/3936), HRP-conjugated anti-mouse IgG (1:3000, 7076), HRP-conjugated anti-rabbit IgG (1: 3000, 7074), SMURF2 (1:1000, 12024), β-actin (1:3000, 3700), acetylated lysine antibody (1:1000, 9441), DYKDDDDK (same as Flag, 1:3000, 14793), V5 (1:2000, 80076). Anti-RACK1 antibodies (1:1000, 66940-1-Ig), Anti- eIF3a antibodies (1:1000, 67713-1-Ig) and Anti- EIF3C antibodies (1:1000, 12733-1-AP) were purchased from Proteintech.

Drugs tested in the study, including Z-Leu-Leu-Leu-al (MG132; HY-13259), cycloheximide (CHX; HY-12320), trichostatin A (TSA; HY-15144) and nicotinamide (NAM; HY-B0150) were purchased from MedChemExpress (MCE). The drugs were dissolved in dimethyl sulfoxide (DMSO). Cells were plated in six-well plates prior to drug treatment and treated with the indicated drugs at the concentrations described in the corresponding figure legends for varying lengths of time when the cells reached 60% confluence. After treatment, cells were harvested for protein extraction and IB analysis. Anti-HA magnetic beads (HY-K0201A), anti-His magnetic beads (HY-K0209), anti-c-Myc magnetic beads (HY-K0206) and protein A/G magnetic beads (HY-K0202) were also purchased from MCE.

### Cell culture and tissue samples

HEK293T, human OC cells SKOV3, Caov-3, A2780, HEY, SW626, Anglne and OVCAR3 were provided by Procell Life Science & Technology Co. (Wuhan, China). HOSE and IOSE80 were provided by the Stem Cell Bank of the Chinese Academy of Sciences. All cell lines were free of mycoplasma and identified by short tandem repeat (STR) DNA fingerprinting at Procell Life Sciences & Technology LTD. All cell lines were grown at 37 °C in 5% CO2 in their respective complete medium containing 10% FBS. With patient consent, 15 cases of fresh OC specimens and 11 cases of fallopian tubal tissues were obtained from Harbin Medical University Cancer Hospital. The FT specimens were obtained from patients diagnosed with gynecological benign tumors who had undergone hysterectomy and bilateral salpingectomy.

### Protein expression and purification

For the production of proteins from bacteria, Escherichia coli BL21 (DE3) cells containing the GST, GST-SMURF2 and GST-SMURF2 C716A plasmids were induced for protein expression using 0.5 mM IPTG at 37 °C for 4–6 h. The cells were lysed in buffered saline. Cell lysis was performed with lysis buffer (0.5% Triton X-100, pH 7.5, 1 mM DTT, 50 mM Tris-Cl, 200 mM NaCl, 10% glycerol and 1 mM PMSF and sonicated). Lysates were centrifuged and incubated with glutathione-Sepharose 4B (GE Healthcare) at 4 °C for 4 h or overnight. The resin was washed three times with lysis buffer plus 300 mM NaCl and then washed two more times with PBS. Immobilization on glutathione-Sepharose beads was verified by SDS-PAGE and aliquoted for storage at −80 °C. 6×His-RACK1 was purified using nickel-nitrilotriacetic acid (Ni-NTA) matrices (QIAGEN).

### In vivo ubiquitination assay

Cells were transfected with the indicated plasmids, then treated with 20 μM MG132 for 8 h for the in vivo RACK1 ubiquitylation assay. The cells were harvested and lysed in RIPA lysis buffer plus 1% SDS, 20 µM MG132, and protease inhibitors. The lysates were incubated with anti-RACK1 or anti-Myc antibodies for 12 h and with Protein A/G magnetic beads for an additional 12 h at 4 °C. The precipitated protein was boiled in SDS-PAGE loading buffer for 10 min and then treated with IB.

### GST pull-down assay

Bacterially expressed GST, GST-SMURF2, or GST-SMURF2 C716A was bound to glutathione-Sepharose 4B beads (GE Healthcare). The complexes were mixed with Myc-RACK1 expressed in HEK293T cells for 2 h at 4 °C. After incubation, the complexes were washed with GST binding buffer for at least 3 times, then eluted with SDS-PAGE loaded buffer by boiling and treated with IB antibody shown.

### Statistics and reproducibility

GraphPad Prism (version 8.0) was used for all statistical analyses. All in vitro experiments were carried out in at least three replicates and the data presented are from one representative experiment. The data are expressed as mean ± standard deviation. Double-tailed Student’s t-test or two-factor analysis of variance was used to assess the statistical significance of differences. The correlation between SMURF2 and RACK1 expression in OC patients was calculated by Pearson correlation analysis. Overall survival was assessed by the Kaplan-Meier method and compared by the log-rank test. *P* < 0.05 was considered statistically significant.

### Supplementary information

Supplementary Figure legends

Figure S1

Figure S2

Figure S3

Figure S4

Figure S5

Supplementary Table1.

Supplemental Materials and Methods.

Original western blot legends

## Data Availability

Mass spectrometry data was uploaded in the Supplementary Table 1. Other datasets are available from the corresponding author on reasonable request.
