# Supplementary material for: Loss of SMURF2 expression enhances RACK1 stability and promotes ovarian cancer progression
Source: Cell Death Differ. 2023 Oct 12;30(11):2382–92. doi: 10.1038/s41418-023-01226-w (PMC10657365; doi:10.1038/s41418-023-01226-w)
Supplement: Supplementary file 1 — Supplementary Figure legends [file 41418_2023_1226_MOESM1_ESM.docx]

**Supplementary Figure legends**

**Supplementary Figure 1. SMURF2 interacts with RACK1.**

(A) Kaplan-Meier plot of overall survival based on RNA sequencing results from TCGA and GTEx databases. Statistical significance was calculated using the log-rank test. (B) SMURF2 expression was down-regulated in OVCAR3 cell lines with two independent shrnas to detect potential target protein expression of SMURF2. (C) Cell lysates from A2780 were analyzed by IP using antibodies against SMURF2 and RACK1, then subjected to IB analysis. (D-E) HA-SMURF2 and Myc-RACK1 were co-transfected into HEK293T cells. The cell lysates were subjected to IP with anti-HA or anti-Myc antibodies. (F) Schematic representation of Myc-tagged FL RACK1 and its various deletion mutants. (G) HEK293T cells were cotransfected with HA-SMURF2 and Myc-tagged FL RACK1 or its deletion mutants, and cell lysates were analyzed by IP with HA beads followed by IB with antibodie against Myc. (H) The confocal image above shows SMURF2(green) and RACK1(red) co-located in SKOV3 (up panel). HEK293T was co-transfected with the plasmids HA-SMURF2 and Myc-RACK1 for 24h. Confocal image showing HA (green) and Myc(red) co-located in HEK293T (below). Nuclei were counterstained with DAPI (blue). Scale bars: 10 μm (H).

**Supplementary Figure 2.** **SMURF2 negatively regulates RACK1 stability.**

(A) Three independent shRNAs of SMURF2 were validated by IB in OVCAR3 cells. The expression efficiency of SMURF2 WT and SMURF2 C716A plasmids was validated by IB in SKOV3 and A2780 cells. (B) In SKOV3 and A2780 cells, RACK1 mRNA levels were detected by qRT-PCR after SMURF2 was ectopically overexpressed. (C) OVCAR3 cells transfected with 2 independent SMURF2 shRNA were treated with or without the autophagy inhibitor CQ (25 μM, 24h), and then SMURF2 and RACK1 were analyzed.

**Supplementary Figure 3. SMURF2 is an E3 ubiquitin ligase for RACK1.**

(A) In vitro RACK1 ubiquitination linkage assay. Purified 6 × His-RACK1 was incubated with or without HA-SMURF2 in the presence of essential components of ubiquitination system including HA-RBX1, Myc-Cullin 3, E1, UbcH5C, UBE1, and WT or mutant ubiquitin for 2 h at 37 °C. After reaction, RACK1 was immunoprecipitated with anti-RACK1 antibody and immunoblotted with anti-ubiquitin antibody. (B) RACK1 poly-ubiquitination linkage was examined by transfecting His-tagged WT or indicated ubiquitin mutants containing point mutations of lysine 6, 11, 27, 29, 33, 48, or 63 to arginine together with Myc-RACK1 into HEK293T cells, followed by IB analysis of His-Ub in anti-Myc IP products. Cells were treated with 20 μM MG132 for 8 h.

**Supplementary Figure 4.** **RACK1 acetylation inhibits interaction with SMURF2, resulting in RACK1 stabilization.**

(A) SKOV3 cells were cotransfected with SMURF2 and Myc-RACK1 or Myc-RACK1 K130Q, treated with 100 μg/ml CHX, collected at the indicated times, and then subjected to IB with antibodies against SMURF2 and Myc. Quantification of Myc levels relative to β-actin is shown. (B) A2780 cells were cotransfected with SMURF2 and Flag-vector or Flag-PCAF, treated with 100 μg/ml CHX, collected at the indicated times, and then subjected to IB with antibodies against SMURF2 and RACK1. Quantification of RACK1 levels relative to β-actin is shown. (C) Ubiquitination assay of RACK1 in SKOV3 cells cotransfected with His-Ub, HA-SMURF2, Myc-RACK1 WT and Flag-PCAF and treated with 20 μM MG132 for 8 h.

**Supplementary Figure 5. Loss of SMURF2 promotes ovarian tumorigenesis via the upregulation of RACK1.**

(A) Three independent shRNAs and overexpression plasmid of RACK1 were validated by IB in A2780 cells. (B) RACK1 was overexpressed ectopic in SKOV3 cells with high SMURF2 expression. Cell migration was examined by transwell assay (B). Scale: 200μm (B). (C) RACK1 was knocked down in OVCAR3 cells with low SMURF2 expression. Cell growth was examined by colony formation (C) Scale: 1 cm (C). B, C Results are representative of three independent experiments, the data are shown as mean ± s.d. One-way ANOVA test, ***p < 0.001.
