# Supplementary figures and images for: Loss of SMURF2 expression enhances RACK1 stability and promotes ovarian cancer progression
Source: Cell Death Differ. 2023 Oct 12;30(11):2382–92. doi: 10.1038/s41418-023-01226-w (PMC10657365; doi:10.1038/s41418-023-01226-w)

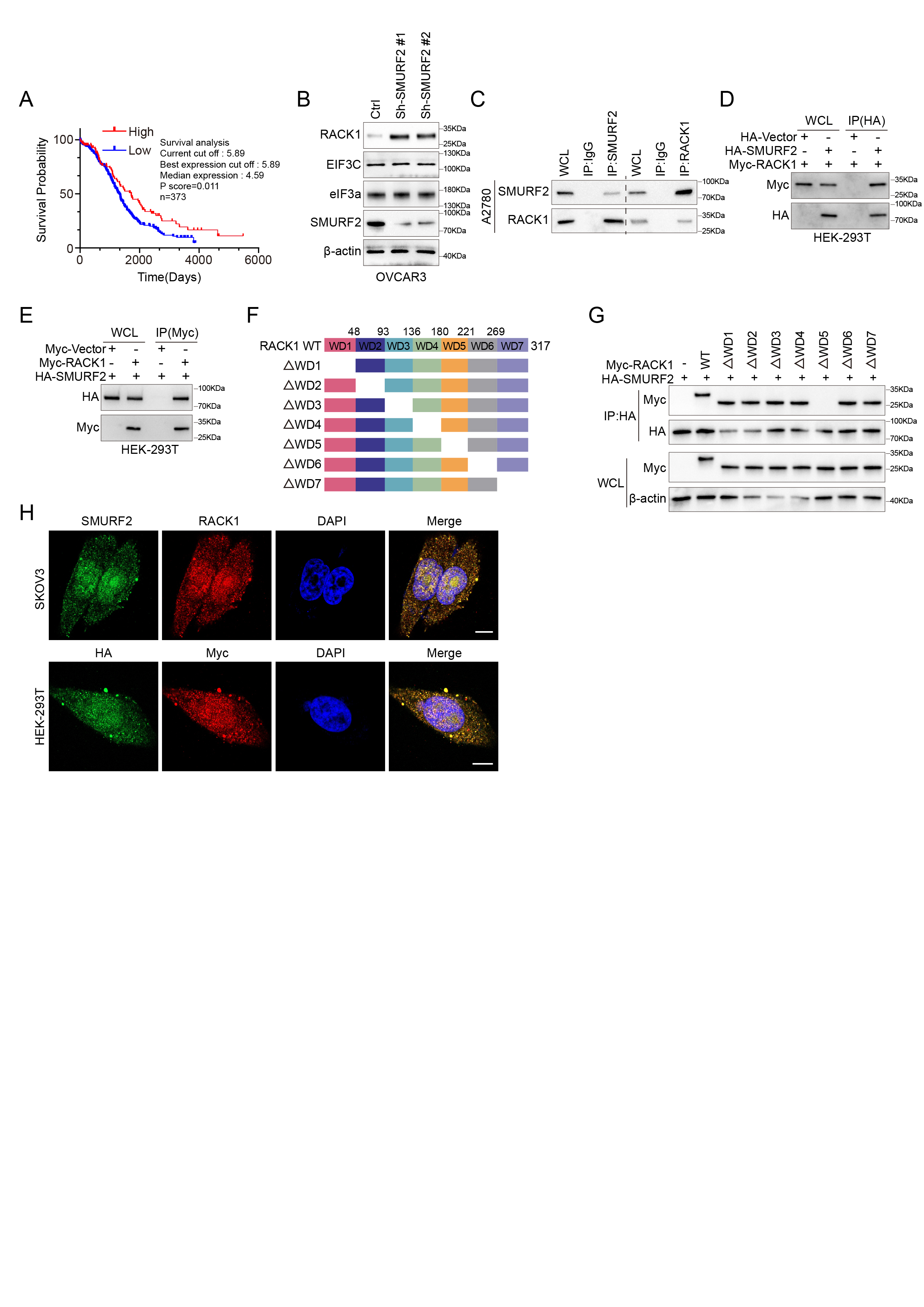

Supplement: Supplementary file 2 — Figure S1 [file 41418_2023_1226_MOESM2_ESM.jpg]

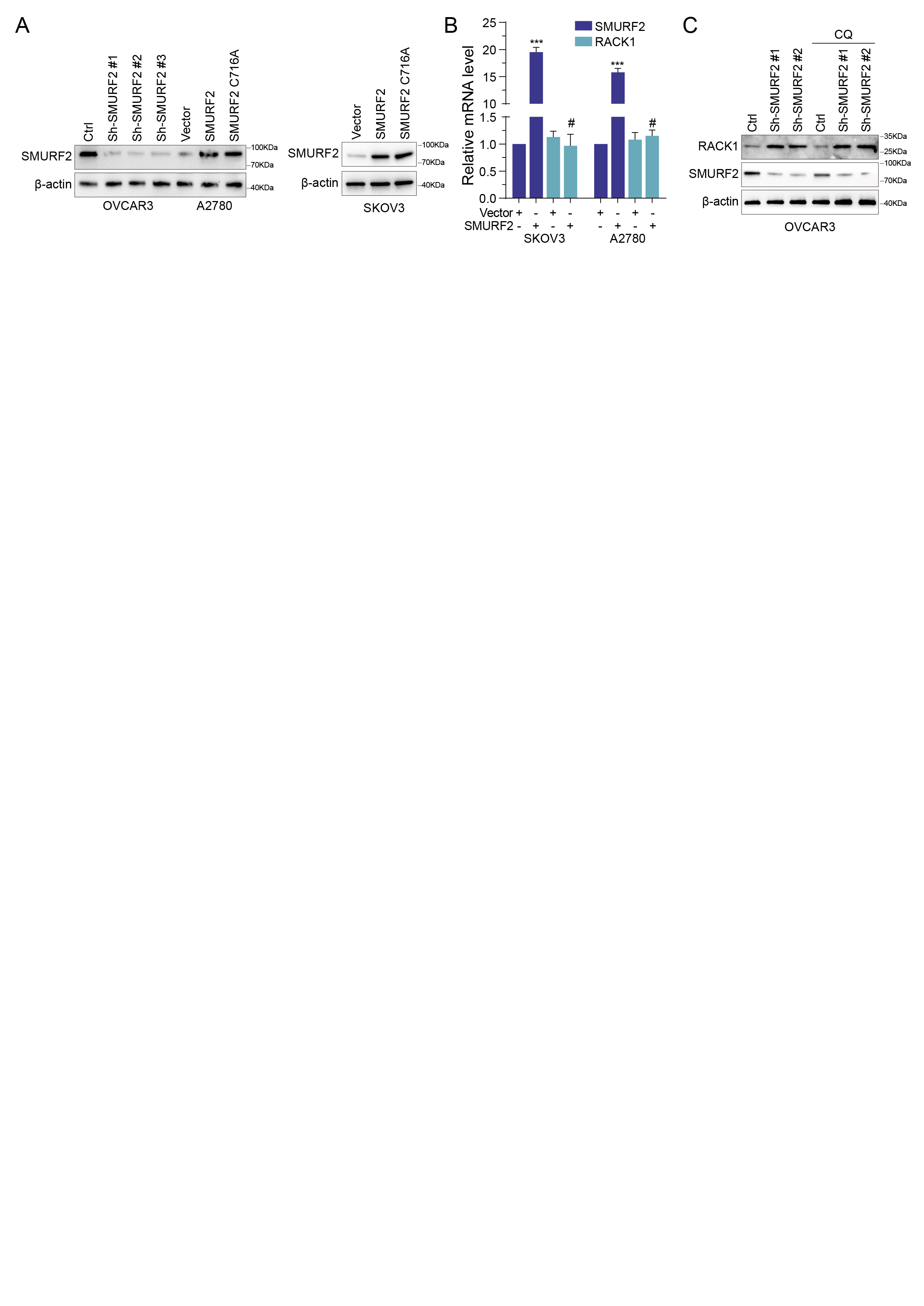

Supplement: Supplementary file 3 — Figure S2 [file 41418_2023_1226_MOESM3_ESM.jpg]

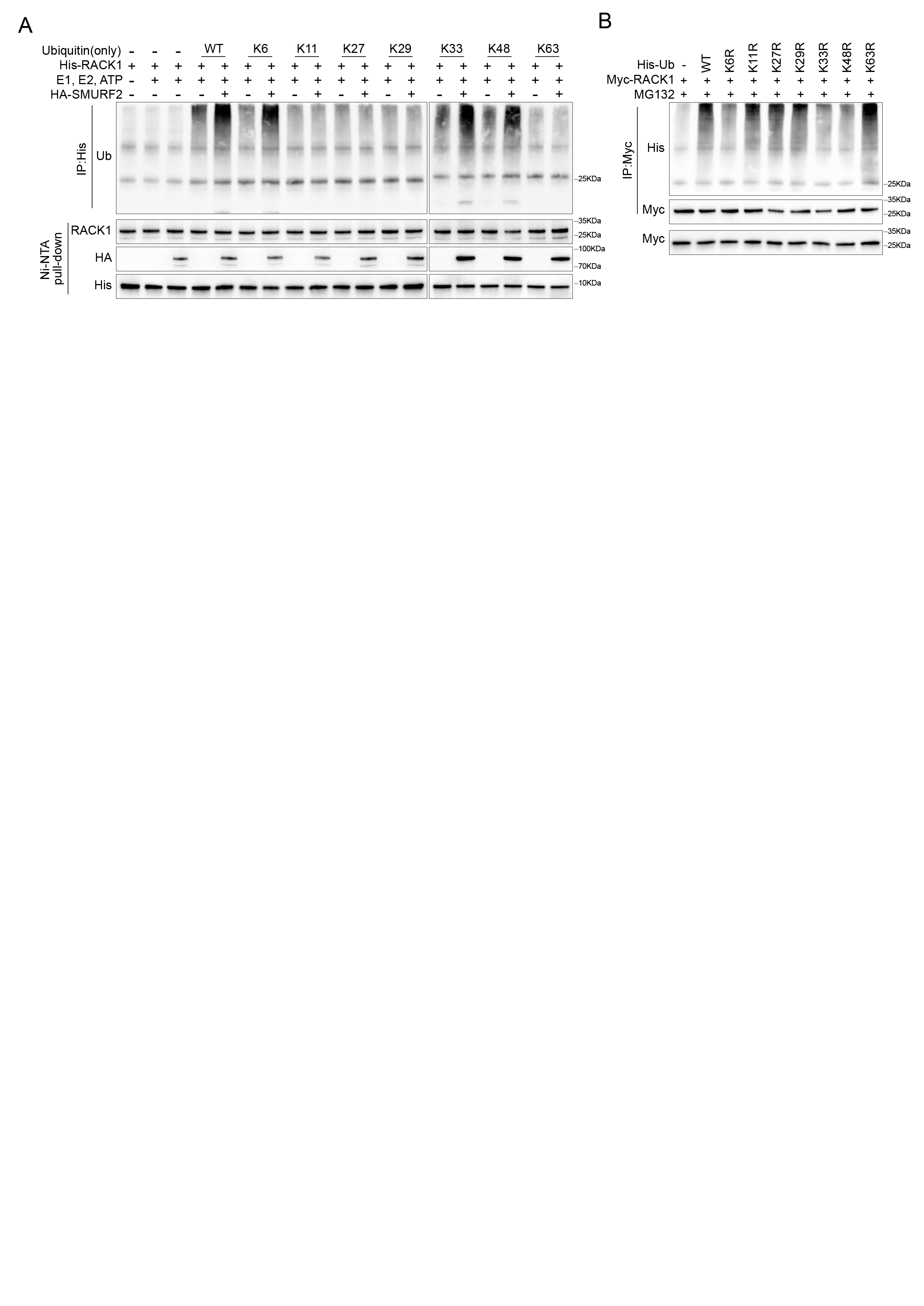

Supplement: Supplementary file 4 — Figure S3 [file 41418_2023_1226_MOESM4_ESM.jpg]

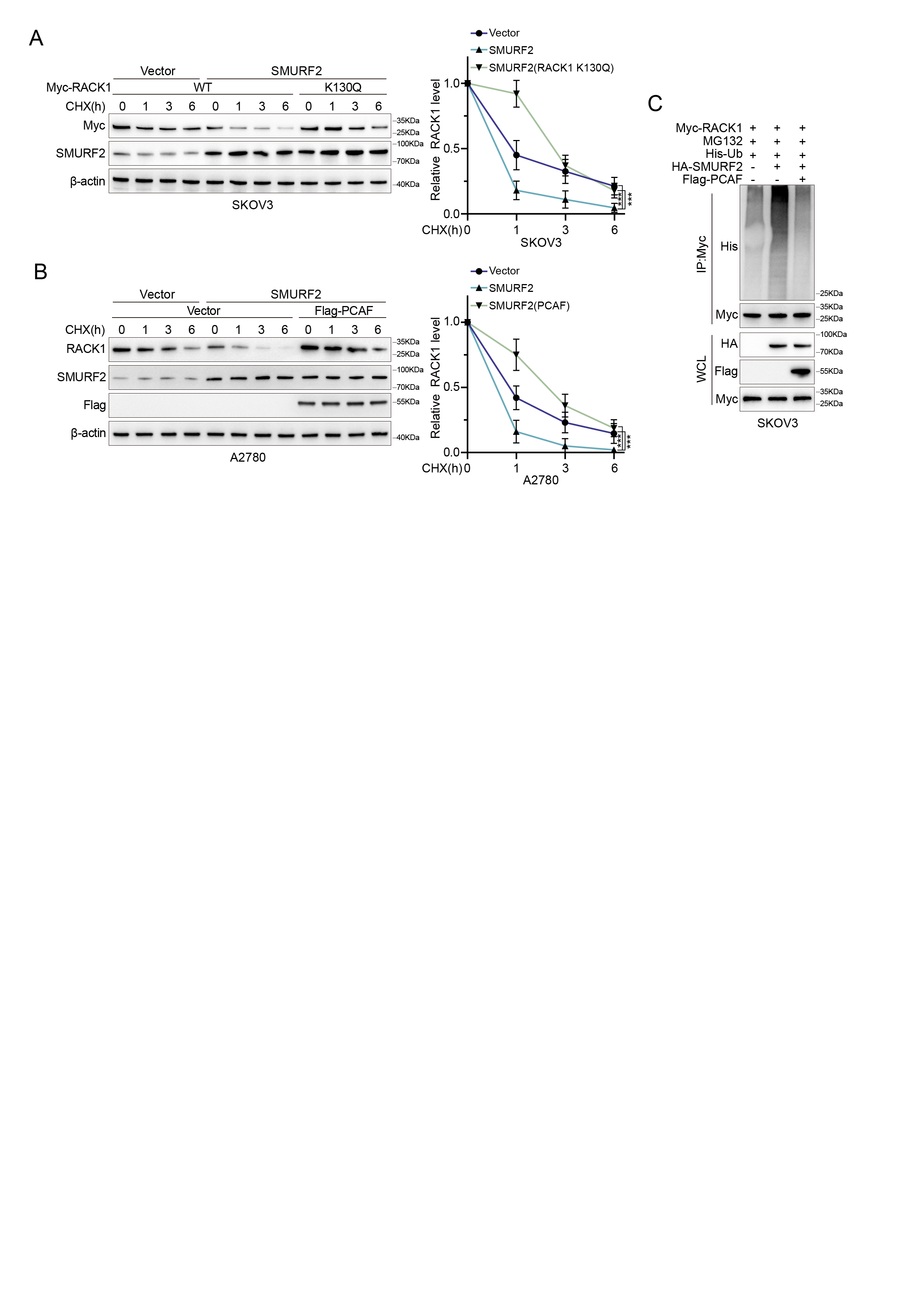

Supplement: Supplementary file 5 — Figure S4 [file 41418_2023_1226_MOESM5_ESM.jpg]

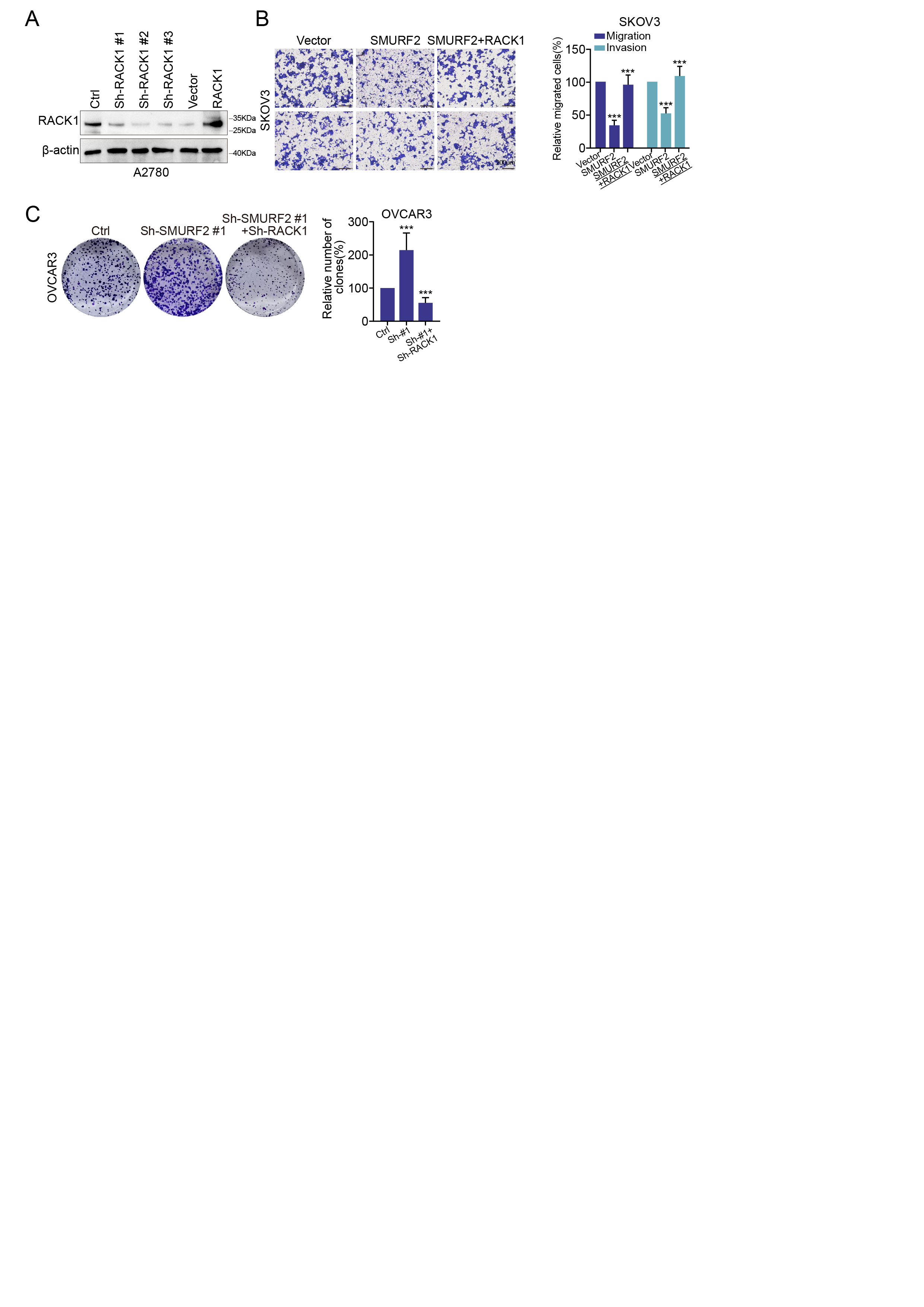

Supplement: Supplementary file 6 — Figure S5 [file 41418_2023_1226_MOESM6_ESM.jpg]

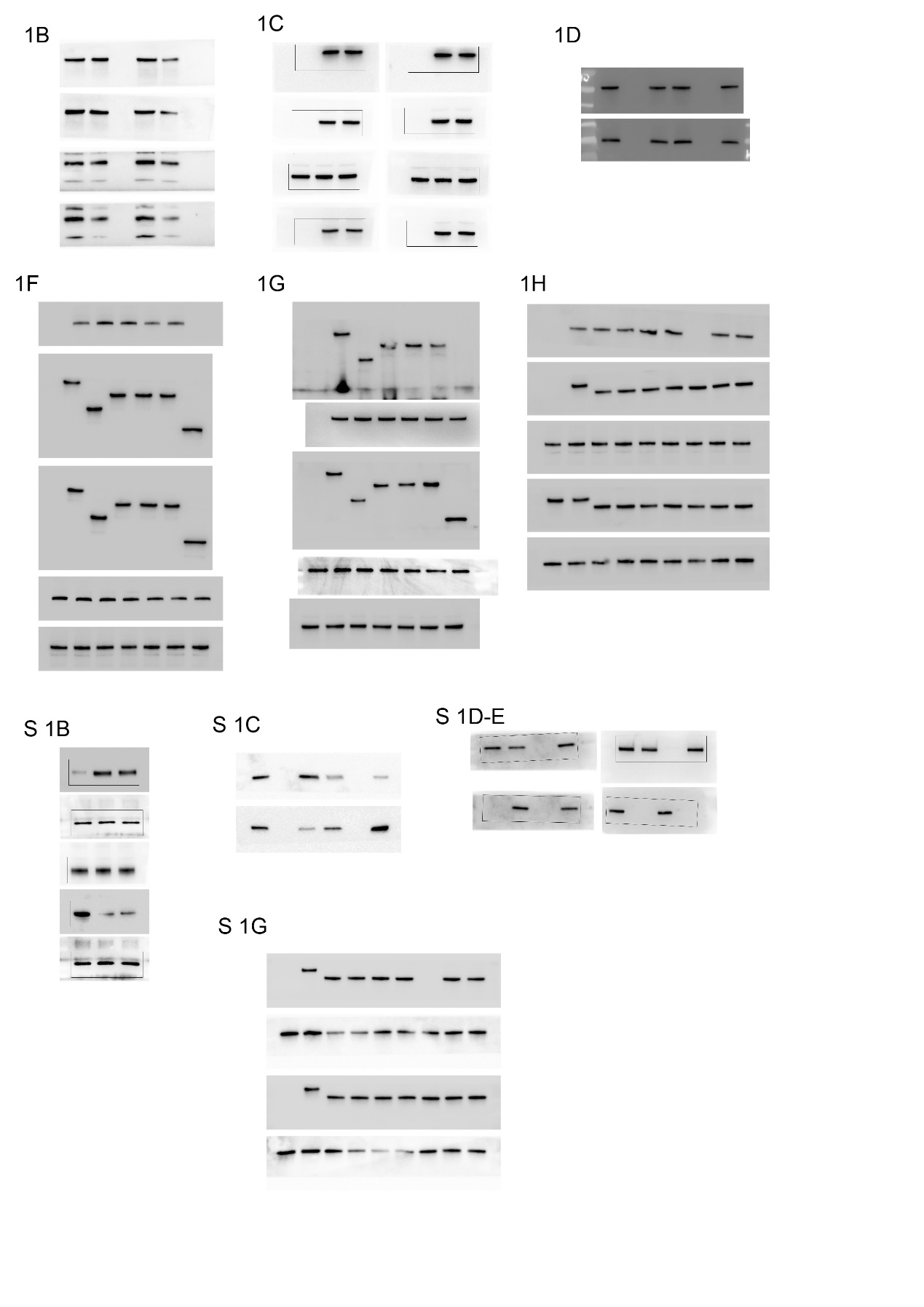


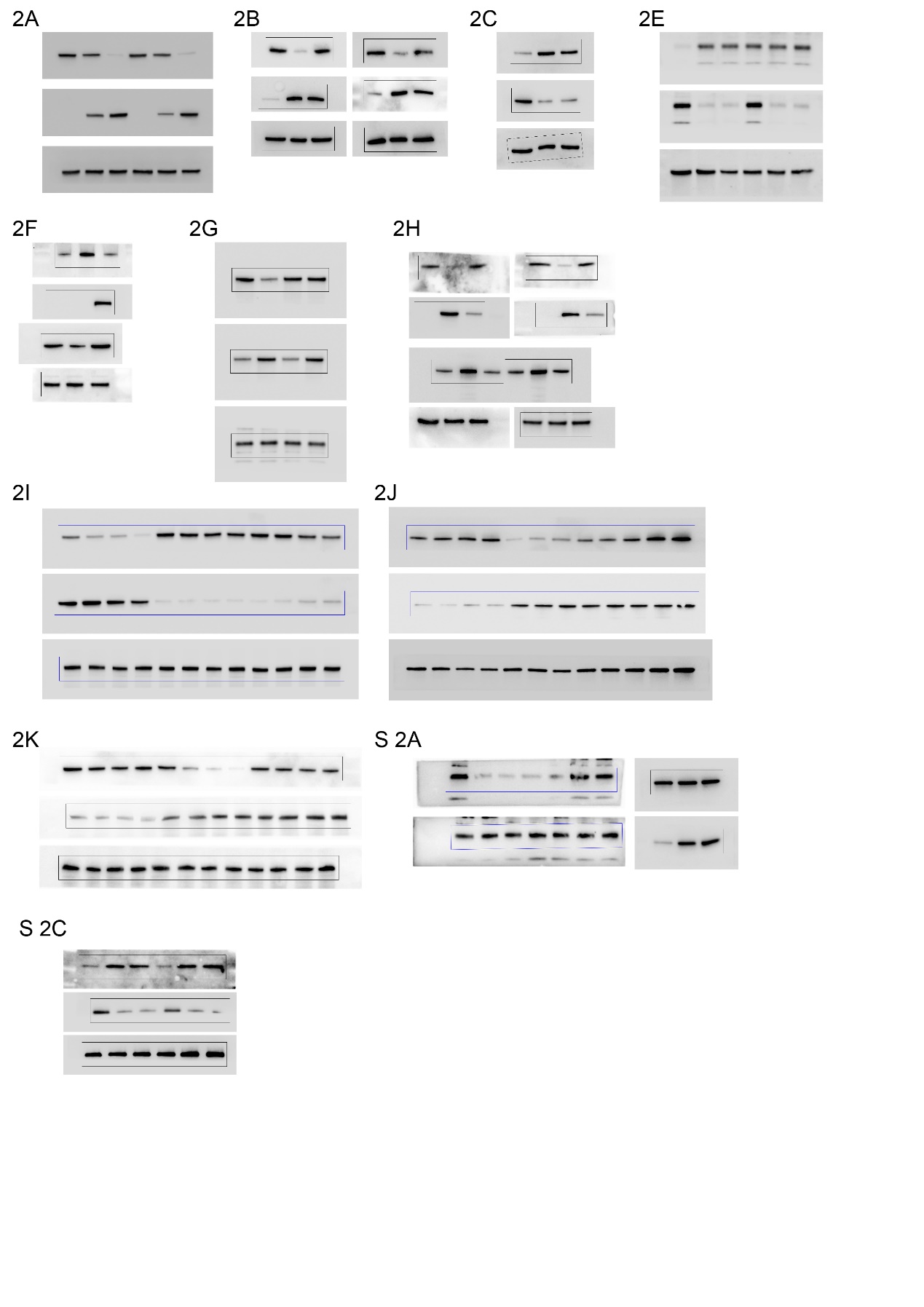


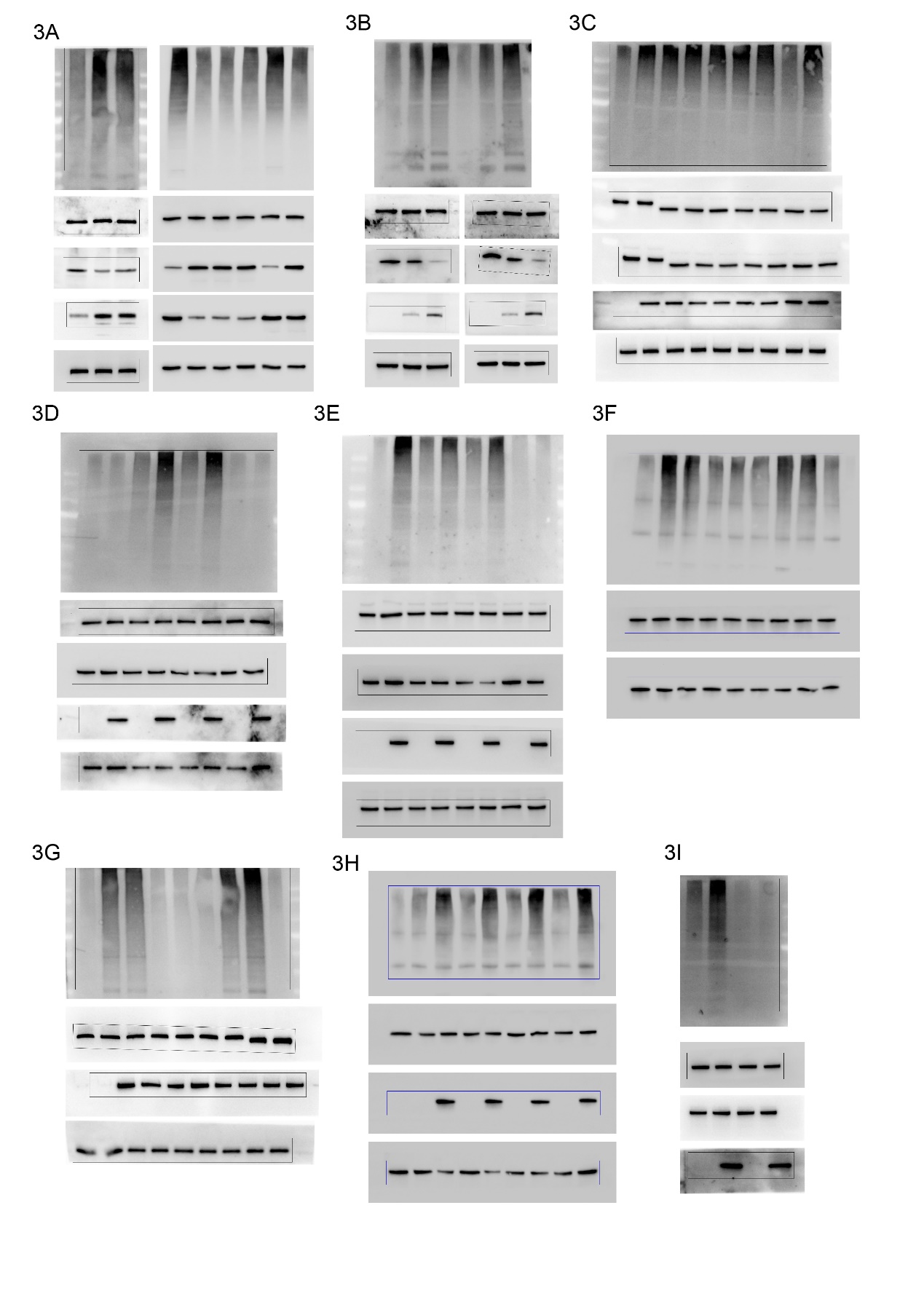


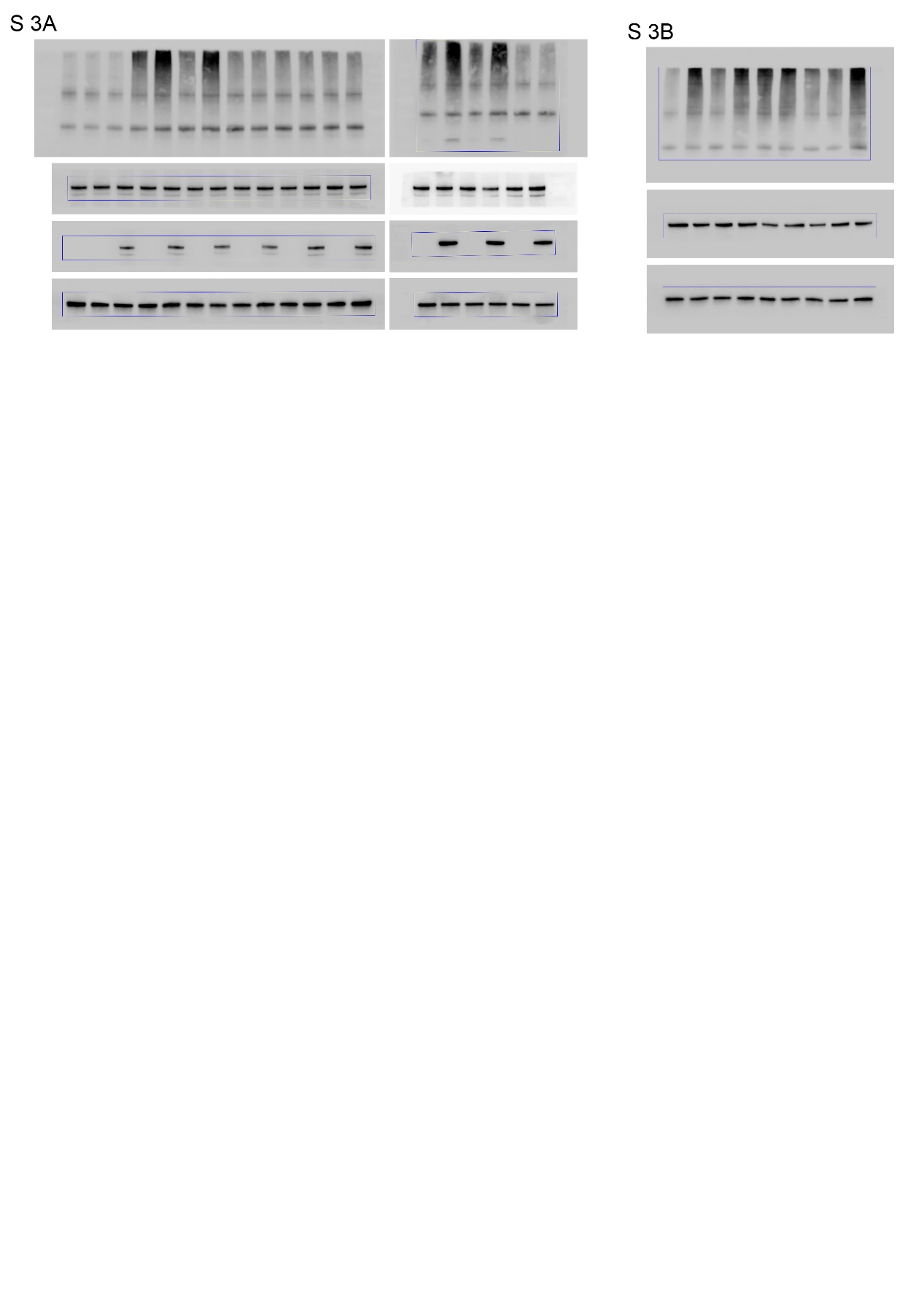


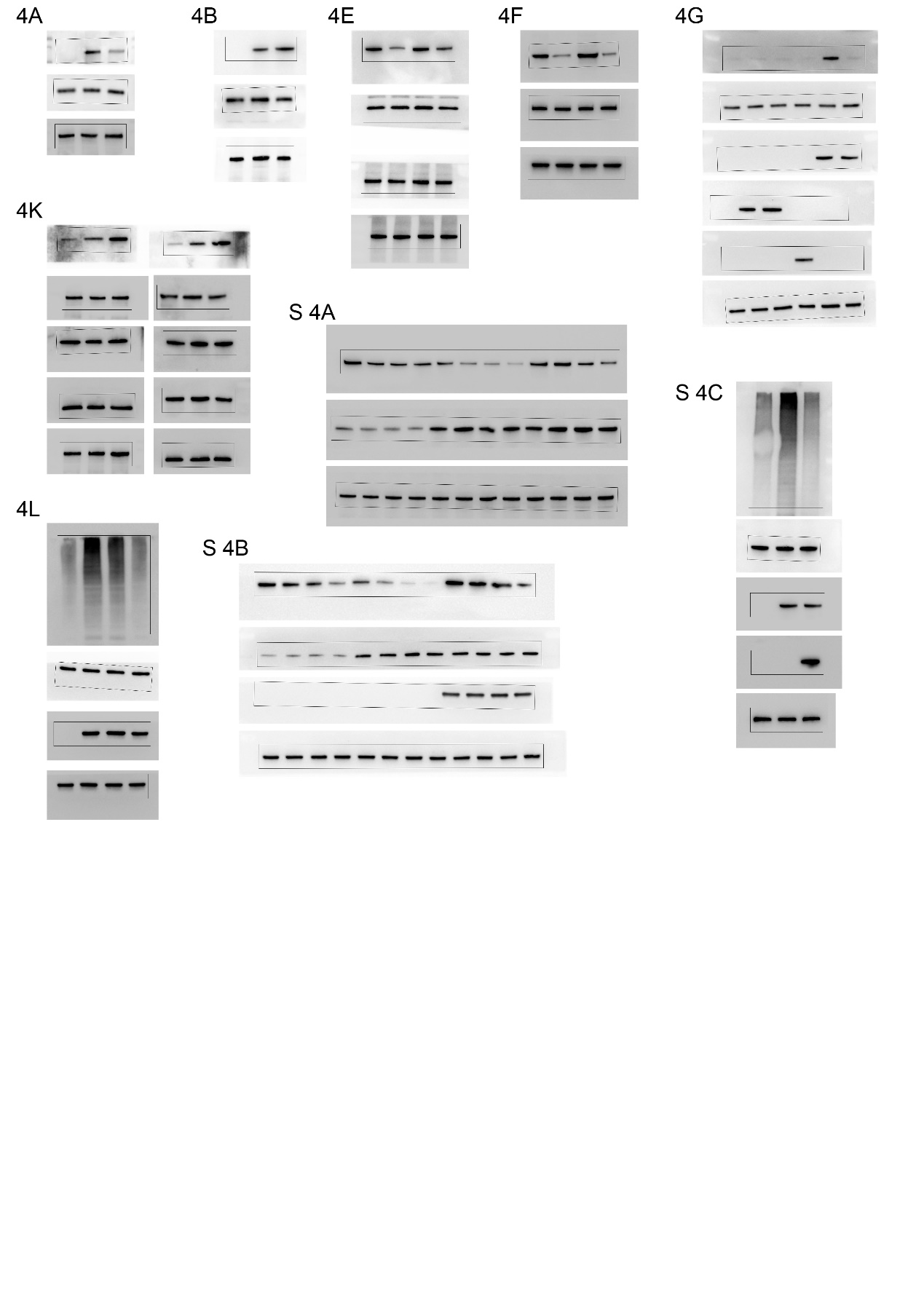


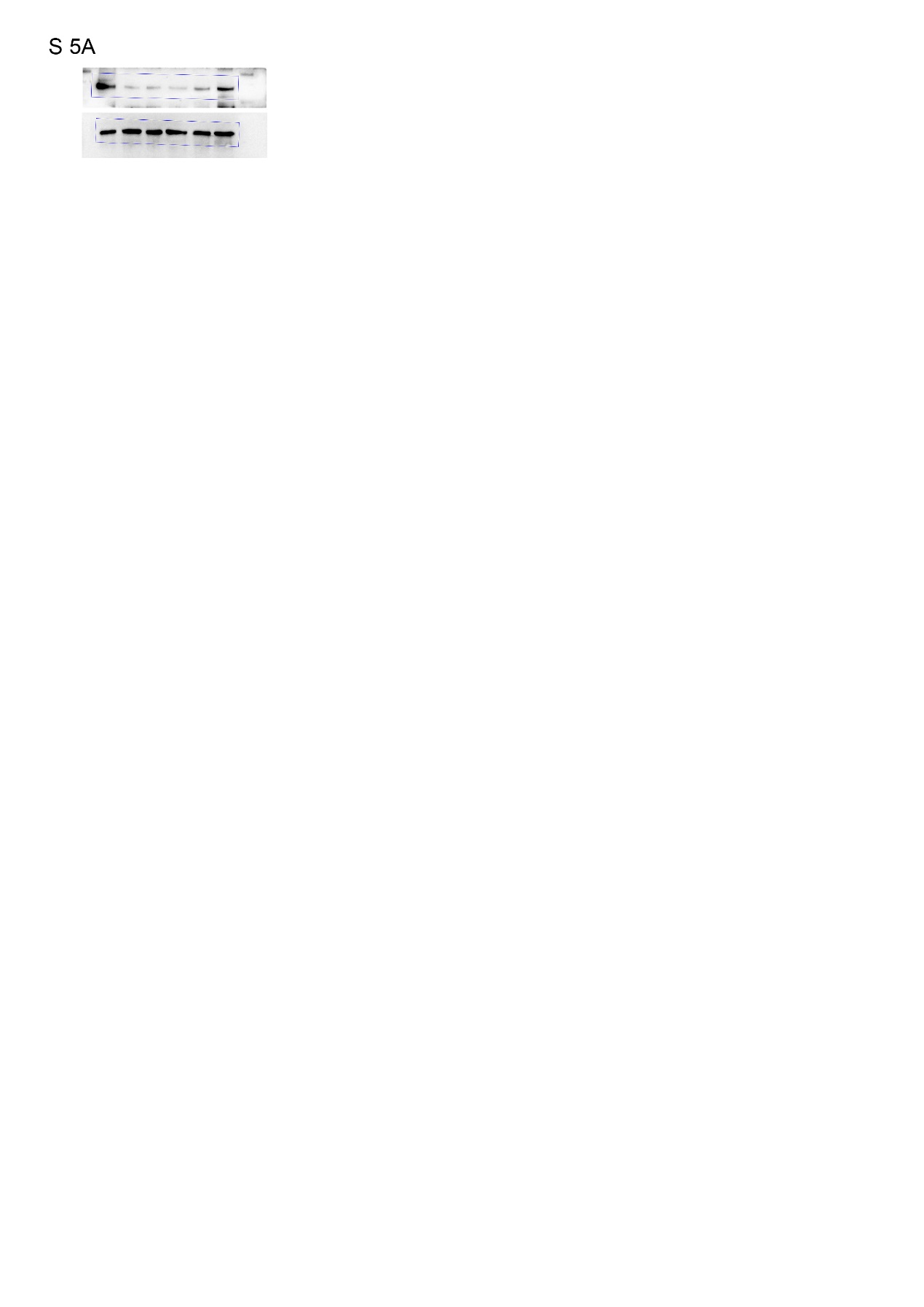


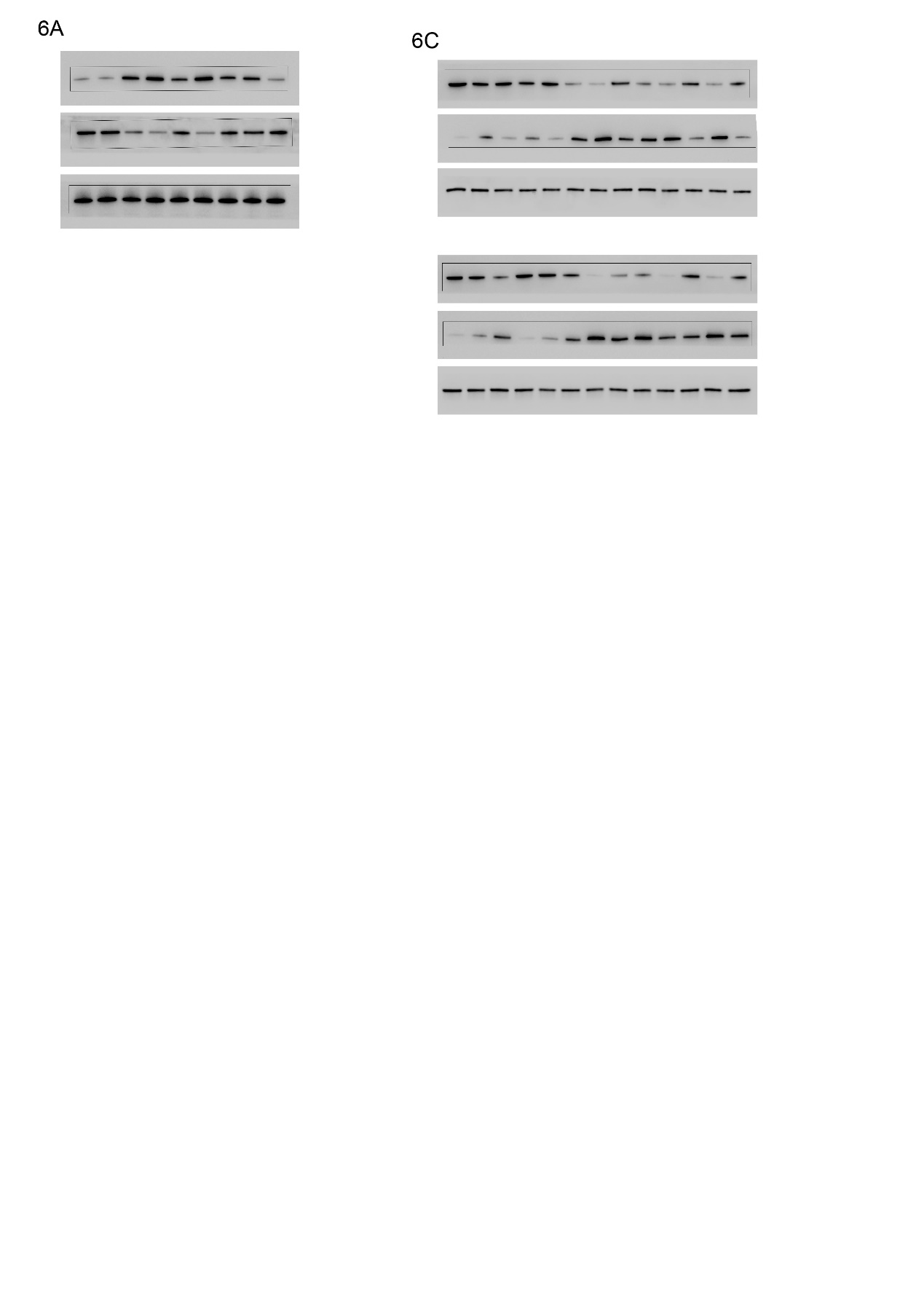

Supplement: Supplementary file 9 — Original western blot legends [file 41418_2023_1226_MOESM9_ESM.docx]
