# Supplementary material for: Loss of SMURF2 expression enhances RACK1 stability and promotes ovarian cancer progression
Source: Cell Death Differ. 2023 Oct 12;30(11):2382–92. doi: 10.1038/s41418-023-01226-w (PMC10657365; doi:10.1038/s41418-023-01226-w)
Supplement: Supplementary file 8 — Supplemental Materials and Methods. [file 41418_2023_1226_MOESM8_ESM.docx]

**Supplementary materials and methods**

**Plasmids.**

HA-SMURF2 and HA-SMURF2 C716A were prepared by cloning human SMURF2 cDNA into the pcDNA3-HA vector. Myc-RACK1 was generated by cloning human RACK1 cDNA into the pcDNA3-Myc vector. His-RACK1 was produced by cloning of human RACK1 cDNA into the pcDNA3-His vector. HA-P300 and HA-CBP were generated by cloning human P300 cDNA and CBP cDNA, respectively, into the pcDNA3-HA vector. Flag-PCAF and Flag-GCN5 were generated by cloning human PCAF cDNA and GCN5 cDNA, respectively, into the pcDNA3-Flag vector. V5-Tip60 was generated by transferring human Tip60 cDNA into the pcDNA3-V5 vector.

Lentiviral vectors expressing SMURF2 or RACK1 were prepared by cloning SMURF2 cDNA or RACK1 cDNA into the pCDH-EF1-FHC vector. SMURF2 shRNA 1, SMURF2 shRNA 2, SMURF2 shRNA 3, RACK1 shRNA 1, RACK1 shRNA 2, and RACK1 shRNA 3 were provided by Zhou Shuai. By cloning the corresponding human cDNA into pGEX-6P-1, the GST-SMURF2 (full length), GST-SMURF2 C716A constructs were generated.

Point mutations were generated by site-directed mutagenesis. The Myc-RACK1 mutants K225R, K257R, K130/172R, K130R, K130Q, K172R, K225R/K257R/K264R (Myc-RACK1 3KR), K225R/K257R (Myc-RACK1 K264), K225R/K257R (Myc-RACK1 2KR), K257R/K264R (Myc-RACK1 K225), K225R/K264R (Myc-RACK1 K257), were generated using Myc-RACK1 as a template. His-ubiquitin WT, K6, K11, K27, K29, K33, K48, K63, K6R, K11R, K27R, K29R, K33R, K48R, K63R, K6/33/48R (HA-ubiquitin 3KR) was kindly provided by Zhou Shuai.

**Antibodies and reagents.**

Antibodies were purchased from Cell Signaling Technology against the following proteins. HA (1:2000, 3724), Myc (1:2000, 2276), His (1:2000, 12698), ubiquitin (1:1000, 20326/3936), HRP-conjugated anti-mouse IgG (1:3000, 7076), HRP-conjugated anti-rabbit IgG (1: 3000, 7074), SMURF2 (1:1000, 12024), β-actin (1:3000, 3700), acetylated lysine antibody (1:1000, 9441), DYKDDDDK (same as Flag, 1:3000, 14793), V5 (1:2000, 80076). Anti-RACK1 antibodies (1:1000, 66940-1-Ig) were purchased from Proteintech.

Drugs tested in the study, including Z-Leu-Leu-Leu-al (MG132; HY-13259), cycloheximide (CHX; HY-12320), nicotinamide (NAM; HY-B0150), and trichostatin A (TSA; HY-15144), were purchased from MedChemExpress(MCE). The drugs were dissolved in dimethyl sulfoxide (DMSO). Cells were plated in six-well plates prior to drug treatment. Cells were treated with the indicated drugs at the concentrations described in the corresponding figure legends for varying lengths of time when the cells reached 60% confluence. After treatment, cells were harvested for protein extraction and immunoblotting analysis. Protein A/G magnetic beads (HY-K0202), anti-HA magnetic beads (HY-K0201A), anti-His magnetic beads (HY-K0209), and anti-c-Myc magnetic beads (HY-K0206) were also purchased from MCE.

**Cell culture and tissue samples**

HEK293T, human OC cells SKOV3, Caov-3, A2780, HEY, SW626, Anglne and OVCAR-3 were provided by Procell Life Science & Technology Co. (Wuhan, China). HOSE and IOSE80 were provided by the Stem Cell Bank of the Chinese Academy of Sciences. All cell lines were free of mycoplasma and identified by short tandem repeat (STR) DNA fingerprinting at Procell Life Sciences & Technology LTD. All cell lines were grown at 37°C in 5% CO2 in their respective complete medium containing 10% FBS. With patient consent, 15 cases of fresh OC specimens and 11 cases of tubal tissues were obtained from the Harbin Medical University Cancer Hospital. The FT specimens were obtained from patients diagnosed with gynecological benign tumors who had undergone hysterectomy and bilateral salpingectomy.

**Lentiviral vectors and stable cell lines.**

To produce lentiviral vectors, each lentiviral plasmid was co-transfected with a helper plasmid in HEK293T cells. Rev, Gag and VSVG helper plasmids are used to generate SMURF2 or RACK1 lentiviral vectors. DR8.91 and VSVG helper plasmids are used to generate shRNA lentiviruses. At 72 h post-transfection, virus-rich medium was collected and nuclei were separated at 1000 rpm for 3 min and filtered through a 0.45 µm filter (Millipore). Cells were infected with concentrated lentiviral particles for 20-24 h in the presence of 7.5 µg/ml Polybrene (MCE) and screened with appropriate concentrations of puromycin(Beyotime) for 1 week.

**Stable RNAi knockdown.**

For stable knockdown, cells were infected with lentiviral particles expressing shRNAs and screened in the presence of puromycin. The shRNA sequences were shControl, 5′-TACAAACGCTCTCATCGACAAG-3′; shSMURF2 #1, 5′- AGCGAGACCTGGTTCAGAA-3′; shSMURF2 #2, 5′-TGGAAGAATCCAGTATCTA-3′; shSMURF2 #3, 5′-TGGAAGCGATTAATGATAA-3′; shRACK1 #1, 5′-AACTGCAAGCTGAAGACCA-3′; shRACK1 #2, 5′-GGGATCTCAACGAAGGCAA-3′; shRACK1 #3, 5′-TGGCAGAGCTTTACAAATA-3′.

**RNA isolation and quantitative real-time PCR.**

The RNeasy Mini Kit (QIAGEN) was used for isolation of total RNA. The iScript cDNA Synthesis Kit (Bio-Rad) was then used for reverse transcription. RACK1 expression levels were quantified using the 2-ΔΔCt method. The values were normalized to GAPDH. Primer sequences for RACK1 are shown in Supplementary Table 3.

**Protein expression and purification.**

For the production of proteins from bacteria, Escherichia coli BL21 (DE3) cells containing the GST, GST-SMURF2 and GST-SMURF2 C716A plasmids were induced for protein expression using 0.5mM IPTG at 37°C for 4-6h. The cells were lysed in buffered saline. Cell lysis was performed with lysis buffer (0.5% Triton X-100, pH 7.5, 1mM DTT, 50mM Tris-Cl, 200mM NaCl, 10% glycerol and 1mM PMSF and sonicated). Lysates were centrifuged and incubated with glutathione-Sepharose 4B (GE Healthcare) at 4°C for 4h or overnight. The resin was washed three times with lysis buffer plus 300mM NaCl and then washed two more times with PBS. Immobilization on glutathione-Sepharose beads was verified by SDS-PAGE and aliquoted for storage at -80°C. 6×His-RACK1 was purified using nickel-nitrilotriacetic acid (Ni-NTA) matrices (QIAGEN).

**Immunoblotting(IB) and co-IP assay.**

For Immunoblotting analysis, cells were lysed in lysis buffer (50mM Tris-HCl at pH 7.4, 150mM NaCl, 1% Triton X-100, 1mM DTT, supplemented with protease and phosphatase inhibitor cocktail (MCE)) and sonicated on ice for 20s at 20% amplitude (10s on, 10s off). Cell lysates were separated by SDS-PAGE. Protein concentration was detected by Bradford protein assay (Bio-Rad). Co-IP was conducted by IP with the indicated antibodies. Immunoprecipitates were washed three times in lysis buffer and subjected to SDS-PAGE and IP analysis.

**In vivo ubiquitination assay.**

Cells were transfected with the indicated plasmids and then treated with 20μM MG132 for 8h for the in vivo RACK1 ubiquitylation assay. The cells were harvested and lysed in RIPA lysis buffer plus 1% SDS, 20µM MG132, and protease inhibitors. The lysates were incubated with anti-RACK1 or anti-Myc antibodies for 12h and with Protein A/G magnetic beads for an additional 12h at 4°C. Precipitated proteins were released from beads by boiling in SDS-PAGE loading buffer for 10 min and subjected to IB.

**In vitro ubiquitination assay.**

As previously described, the in vitro ubiquitination assay was performed. Briefly, to purify HA-SMURF2 by HA affinity precipitation, HEK293T cells were transfected with plasmids expressing HA-SMURF2. 6× His-RACK1 was subsequently purified using nickel-nitrilotriacetic acid (Ni-NTA) matrices (QIAGEN). The ubiquitination assay was carried out at 37°C for 2 h in 20 µL reaction buffer (20× 10^−3^ M Tris-HCl; pH 7.2; 5×10^−3^ M MgCl2; 50×10^−3^ M NaCl; 1×10^−3^ M 2-mercaptoethanol; 10% glycerol) containing the following components: 100×10^−9^ M UBE1, 2×10^−6^ M UbcH5a, 4×10^−3^ M ATP, 1×10^−6^ M ubiquitin aldehyde, 50×10^−6^ M ubiquitin WT or K6, or K11, or K27, or K29, orK33, orK48, or K63 (all from Ubiquitin-Proteasome Biotechnologies, Cat# J3220), 5× 10^−6^ m of HA-SMURF2 and His-RACK1. The reaction was terminated by adding 0.4 mL pulldown buffer (20×10^−3^ m Tris-HCl, pH 7.5; 500×10^−3^ M NaCl; 1% Triton X-100; 0.02% BSA; and 5× 10^−3^ M β-mercaptoethanol). After incubation for 8 h at 4°C, 60 µL of anti-His magnetic beads were added. Beads were washed three times with 1mL pulldown buffer. By boiling 50µL of 2× SDS-PAGE sample buffer for 10 min, the proteins bound to the beads were released. The samples were then resolved by 10% SDS-PAGE. IB analysis was then performed.

**GST pull-down assay.**

For the GST pull-down assay, bacterially expressed GST, GST-SMURF2, or GST-SMURF2 C716A was bound to glutathione-Sepharose 4B beads (GE Healthcare). The complexes were mixed with Myc-RACK1 expressed in HEK293T cells for 2 h at 4°C. After incubation, the complexes were washed at least 3 times with GST binding buffer, eluted by boiling in SDS-PAGE loading buffer, and subjected to IB with the indicated antibodies.

**Colony formation and cell migration assays**

For the colony formation assays, A2870, SKOV-3 and OVCAR-3 cells transfected with the indicated genes are cultured at 1000 cells per well in a six-well plate in an incubator at 37°C and 5% CO^2^ for 2 weeks. The colonies were then stained with 0.1% crystal violet and the cells counted. To measure cell motility, 5×10^4^ cells were plated in the upper chamber with the uncoated membrane (24-well insert; pore size, 8 μm; BD Biosciences) in serum-free medium, and serum supplemented medium was used as chemoattractant in the lower chamber. Cells were incubated for 20h and cells not migrating through the pores were removed with a cotton swab. Those in the lower filters were stained with 0.1% crystal violet for 20min and counted.

**Immunohistochemistry (IHC).**

The tissue microarray of OC with clinicopathologic data was purchased from SHANGHAI OUTDO BIOTECH CO., LTD. Immunohistochemical staining for SMURF2 and RACK1 was performed on the tissue microarray. The protein expression in the sample was estimated based on the percentage and intensity of stained tumor cells. The scores for percentage and intensity of staining were as follows: 0 (0-4%), 1 (5-24%), 2 (25-49%), 3 (50-74%), or 4 (≥75%) and 0, 1, 2, or 3, respectively. The final score was multiplied by the percentage and intensity scores. Two investigators, blinded to the clinical information of the OC patients, scored all sections separately.

**Animal experiments**

Animal experiments were performed in accordance with the guidelines approved by the Animal Care and Use Committee of Harbin Medical University Cancer Hospital. Four-week-old female BALB/c nude mice were subcutaneously inoculated with 4×106 cells stably transfected with the indicated lentivirus. Tumor length and width were measured every 3 days and tumor volume was calculated using the following formula: volume = (L*W ^2)/2. For the lung metastasis model, each 4-week-old BALB/c nude mouse was injected intravenously into the tail vein with 1 × 106 cells suspended in 100 μL PBS. Mice were sacrificed six weeks after cell injection. The lungs were excised and fixed in phosphate buffered formalin for analysis. The lungs were then carefully examined for the number of metastatic nodules.

**Protein Half-Life Assays:**

Cells transfected with the designated plasmids were subjected to treatment with the protein synthesis inhibitor CHX (50 μg/ml; MCE) for the indicated periods before harvesting.

**Immunofluorescence (IF)**

For immunofluorescence staining, HEK293T and human OC cells were fixed with 4% formaldehyde, perfused with 0.25% Triton X-100, and blocked with 1% BSA for 1 hour at room temperature. Cells were detected with appropriate primary antibodies. After washing with PBST, cells were exposed to the appropriate Alexa Fluor 488 or Alexa Fluor 594 conjugated secondary antibodies (Abcam) and DAPI (Biosharp). Cells were then visualized by confocal laser scanning microscopy (Zeiss LSM 5).

**Statistics and reproducibility.**

GraphPad Prism (version 8.0) was used for all statistical analyses. All in vitro experiments were carried out in at least three replicates and the data presented are from one representative experiment. The data are expressed as mean ± standard deviation. Double-tailed Student's t-test or two-factor analysis of variance was used to assess the statistical significance of differences. The correlation between SMURF2 and RACK1 expression in OC patients was calculated by Pearson correlation analysis. Overall survival was assessed by the Kaplan-Meier method and comparisons were made by the log-rank test. P value < 0.05 was considered statistically significant.
